# Utilization of an Asynchronous Online Learning Module Followed by Simulated Scenario to Train Emergency Medicine Residents in Mass-Casualty Triage

**DOI:** 10.21980/J89S7Z

**Published:** 2024-07-31

**Authors:** Justin P Delgado, Douglas Spencer, Leah M Bralow

**Affiliations:** * St. Barnabas Health System, Department of Emergency Medicine, Bronx, NY; ^ City University of New York, Sophie Davis School of Medicine, New York, NY

## Abstract

**Audience:**

This is a combined independent study and simulation session designed to teach and drill Mass Casualty Incident (MCI) Triage and is intended for emergency medicine residents at all levels.

**Introduction:**

The training of emergency medicine residents to assume leadership roles in disaster response is important. However, lack of accepted specific educational goals on the national level leads to significant variability between residencies.

**Educational Objectives:**

The purpose of this session is to train EM residents in the use of the Simple Triage and Rapid Treatment (START) and pediatric JumpSTART algorithms for triage in mass casualty incidents (MCIs) using an asynchronous model. By the end of this small group session, learners will be able to: 1) describe START triage for adult MCI victims; 2) describe JumpSTART triage for pediatric MCI victims; 3) demonstrate the ability to apply the START and JumpSTART triage algorithms in a self-directed learning environment; 4) demonstrate the ability to apply the START and JumpSTART triage algorithms in a simulated mass casualty scenario under time constraints; and 5) demonstrate appropriate use of acute life-saving interventions as dictated by the START and JumpSTART triage algorithms in a high-pressure simulated environment.

**Educational Methods:**

This session utilizes an online independent study module that was created *de novo* for this specific purpose by the authors followed by a high-pressure in-person simulation session where learners practice applying the START triage model with multiple simulated patients under time constraint.

**Research Methods:**

Learner feedback was collected after completion of the session. Retention of learning objectives was tested at four months via multiple-choice quiz.

**Results:**

The session was very well received by our residents, who appreciated the opportunity to practice applying START triage under pressure. The average score on the pretest was 49%. Response rates to the post-test were low, but residents scored an average of 73%, indicating a trend towards retention of learning objectives.

**Discussion:**

Overall, the utilization of a *de novo* online learning module followed by simulation proved to be a well-received method of teaching MCI triage to emergency medicine residents. We consider this to be an effective way to train MCI Triage with minimal in-conference time utilization. We plan to implement this training annually to provide our residents with longitudinal reinforcement of this vital skill.

**Topics:**

Mass casualty, MCI, triage, START triage, JumpSTART Triage, disaster, disaster preparedness, disaster curriculum, prehospital, EMS.

## USER GUIDE

[Table t1-9-3-sg1]

**Table t1-9-3-sg1:** 

List of Resources:	
Abstract	1
User Guide	3
Small Groups Learning Materials	7
Appendix A: START Triage Pre-Test Questions and Answers	7
Appendix B: START Slides	11
Appendix C: START Triage Lecture Video	12
Appendix D: START Triage Algorithm	13
Appendix E: START Triage Learning Scenarios for Online Module	15
Appendix F: Multiple Casualty Incident Simulation	20
Appendix G: Simple START Triage Pocket Card	31
Appendix H: Delayed Post-Test Questions and Answers	32

**Learner Audience:**

Interns, Junior Residents, Senior Residents

**Time Required for Implementation:**

This session can be implemented with approximately one hour of asynchronous learner pre-work and one hour of in-person simulation time.

**Recommended Number of Learners per Instructor**:

1–12 learners depending on resource availability and time constraints.

**Topics:**

Mass casualty, MCI, triage, START triage, JumpSTART Triage, disaster, disaster preparedness, disaster curriculum, prehospital, EMS.

**Objectives:**

The scope of this session is broad, encompassing a number of primary and secondary learning objectives.

*Primary Learning Objectives:*

By the end of this session, learners will be able to:

1. Describe START triage for adult MCI victims.
2. Describe JumpSTART triage for pediatric MCI victims.
3. Demonstrate the ability to apply the START and JumpSTART triage algorithms in a self-directed learning environment.
4. Demonstrate the ability to apply the START and JumpSTART triage algorithms in a simulated mass casualty scenario under time constraints.
5. Demonstrate appropriate use of acute life-saving interventions as dictated by the START and JumpSTART triage algorithms in a high-pressure simulated environment.

*Secondary Learning Objectives:*

By the end of this session, learners will be able to:

1. Demonstrate correct diagnosis and treatment of tension pneumothorax.
2. Accurately identify patients requiring decontamination from presumed chemical exposure prior to emergency treatment.
3. Demonstrate appropriate use of CPR in an MCI for a high voltage electrocution.
4. Demonstrate retention of ability to apply the START and JumpSTART triage algorithms in a multiple-choice testing scenario.

### Linked objectives and methods

This educational session takes advantage of technology and adult learning theory to minimize in-conference time through utilization of online, self-directed learning, spaced repetition, and simulation to rapidly solidify knowledge of the START algorithm for MCI triage. By the completion of this session, learners will demonstrate the ability to apply rapidly and accurately the START Triage algorithm under stress in a simulated MCI scenario.

The small group session involves two components:

#### I. Asynchronous START Triage Training Module

An internet curriculum creation and management platform called Moodle (https://moodle.org/Moodle Pty Ltd, West Perth, Australia) was used to build an online module to train EM residents on the use and application of START and JumpSTART Triage in an MCI. The module consists of a pre-test assessment of resident’s baseline START triage knowledge. Next, the learners watch an eight-minute video training on how to perform START and JumpSTART triage. The video was researched, designed, and created *de novo* by the authors for this specific purpose. The video is followed by training cases where learners as first responders are asked to triage a number of patients in an MCI. Learners are given immediate feedback and instruction as they perform the training cases. There is no time constraint on the training cases.

#### II. In-person MCI Simulation

Within one week after completion of the online learning module, learners participate in a simulated Mass Casualty event. The MCI simulation is run with small groups of resident learners. Resident learners complete the MCI drill either individually or are paired up into teams of two and given 30 seconds to evaluate each of six moulage actors representing patients. If space allows, the patients can be spaced out on a field allowing for resident teams to work in tandem without overhearing each other. In this way, up to 12 residents can go through the scenario simultaneously – each team starts on a different patient and works their way through the sequence of patients until they return to their starting patient. The timekeeper calls for residents to rotate every 30 seconds without discussing their findings until all learners are finished with all six patients. After the completion of all six triage patients, the small group of 10–12 learners gathers for debrief and to discuss relevant triage decisions for each case. The debrief and discussion includes feedback to learners by faculty, review of the correct triage category for each MCI patient, and distribution of an associated pocket reference card (see Appendix G). Questions are answered in full during the debrief. The debrief is led by faculty experienced in disaster preparedness. The simulated MCI is run until all learners have completed the simulation.

Our program runs this simulation at an outdoor park, both to provide space for the large area required when working with high numbers of simulated patients and patient care teams and to allow residents to work in an “austere” environment lacking the resources of the simulation center.

### Recommended pre-reading for facilitator

Small group facilitators should have familiarity with the START and JumpSTART triage algorithms as well as ability to apply these algorithms in practice.

### Learner responsible content (LRC)

Strong foundational skills in MCI triage and hospital-based MCI response are fundamental skills for today’s Emergency Physicians.

We created a *de novo* online START Triage training module for our residents. Learners will complete the 1–2 hour online START Triage training module prior to participation in the MCI simulation. See Appendices A–D for materials associated with the online learning module.

### Small group application exercise (sGAE)

After completion of the online learning module, learners participate in a simulated Mass Casualty event. The MCI simulation is run with small groups of resident learners. Resident learners complete the MCI drill either individually or are paired up into teams of two and given 30 seconds to evaluate each of six moulaged actors representing patients. Residents rotate every 30 seconds without discussing their findings until all learners are finished with all six patients. After the completion of all six triage patients, the small group of 10–12 learners gathers for debrief and to discuss relevant triage decisions for each case. The debrief and discussion includes review of the correct triage category for each MCI patient, individual learner feedback, and distribution of an associated pocket reference card (Appendix G). The debrief is led by faculty experienced in disaster preparedness. The simulated MCI is run until all learners have completed the simulation. Please see Appendix D, F, and G for simulation materials.

**Brief wrap up (optional):** Four months after the simulation event, knowledge was re-tested using a post-test evaluation (see Appendix H).

### Results and tips for successful implementation

Forty-two learners completed both the online learning module and the MCI simulation. Four months after completion of the session, our learners were given a 10-Question online post-test.. The post test is designed to test knowledge and application of the START and JumpSTART triage methods but does not replicate the cases from previous sessions. The post-test is given in an exam format. Learners may review answer choice feedback online only after final submission of the graded exam.

The average pretest score for our learners was 49%. After the online learning module, the average score for the included assessment scenarios was 68%. Four months after the in-person MCI simulation, 14 of the original 42 learners voluntarily completed the post-test. The average score on the reassessment quiz was 73%, suggesting a trend towards knowledge retention after the in-person simulation.

Learners provided feedback on the experience using a 10-point Likert scale, with 10 being the highest score. The quality of the online START Triage learning module received an average score of 9.1 out of 10. Triage module ease-of-use received an average of 9.5. The quality of the in-person MCI simulation was rated 9.3 out of 10.

Initial feedback from learners on both the online learning module and MCI simulation was overwhelmingly positive. Some of the free form comments included, “I thought the module was good preparation for simulation and it was an effective teaching method.” “I really enjoyed the MCI training at Orchard [Beach] and the [online] module definitely helped. I, at the very least, understand how to triage patients in an event.” “[The scenarios] made us think about re-triaging someone after an intervention.” “Great job guys!! The props really helped. Fun way to break up otherwise dry lectures and enjoy some fresh air.”

A common criticism provided by our learners was that our residents wished that they had more than six patients to triage during the simulated in-person MCI. They also requested a deeper educational dive into the management of MCI patients beyond only triage by including hospital-based care. In response to this feedback, we have modified the simulation portion of the session to provide a larger MCI group and more hospital-based clinical education.

We suggest a number of strategies for successful implementation.

- The online learning module is best executed using an online Learning Management System. This allows for mixed learning techniques – exam, video lecture and self-testing – to be combined into a single unified learning module.
- This curriculum can be implemented at any time of year; however, the MCI simulation requires a large amount of space and is best conduced in a low-resource environment, such as a field, to replicate prehospital conditions. We utilize a local public park.
- Many actors are necessary to be simulated patients. We run this curriculum during months when we have a large number of medical students in our department who can be used as actors.
- Designate a faculty member or senior resident as official “timekeeper” during the simulation session. The timekeeper will call for learners to rotate to the next patient when the allotted triage time is up.
- Allow adequate time for the drill to be repeated multiple times until all learners have completed practicing MCI Triage in a high-pressure environment.

### Associated Content

Appendix A: START Triage Pre-Test Word document or available here: https://docs.google.com/forms/d/1cAMMjVVgSTN9pE0j0UxTxiT1LgPZBXzUIKP9D3flnM8/edit?usp=sharing_eip_m&ts=66a5bc89
Appendix B: START Slides
Appendix C: START Triage Lecture Video
Appendix D: START Triage Algorithms
Appendix E: START Triage Learning Scenarios for Online Module Word document or available here: https://docs.google.com/forms/d/1B6V1YaDaVOy6tXNlyewswVrlFEkMsWhYI-NKoankGyY/edit?ts=66a5bc80
Appendix F: Multiple Casualty Incident (MCI) Simulation
Appendix G: Simple START Triage Pocket Card.
Appendix H: 3-months Delayed Post-Test Word document or available here: https://docs.google.com/forms/d/1nv6wKCLZUWPc2rYJs9PN7c8eg8_D9-rR9cFnYj_SNH4/edit?ts=66a5bc78#settings

*Note: To use the Google Forms, first “Make a Copy” so that you have a copy of the form for which you are the owner and can see the responses.

### Pearls

At the end of this session, learners can be provided with printable resource materials for review and reference (Appendix D and G).

## Supplementary Information





## Figures and Tables

**Figure f5-9-3-sg1:**
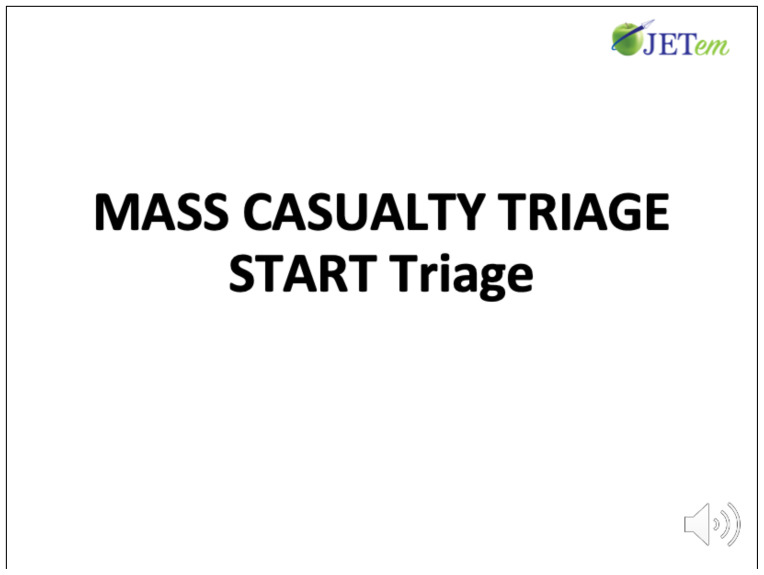
Please see associated PowerPoint file

**Figure f6-9-3-sg1:**
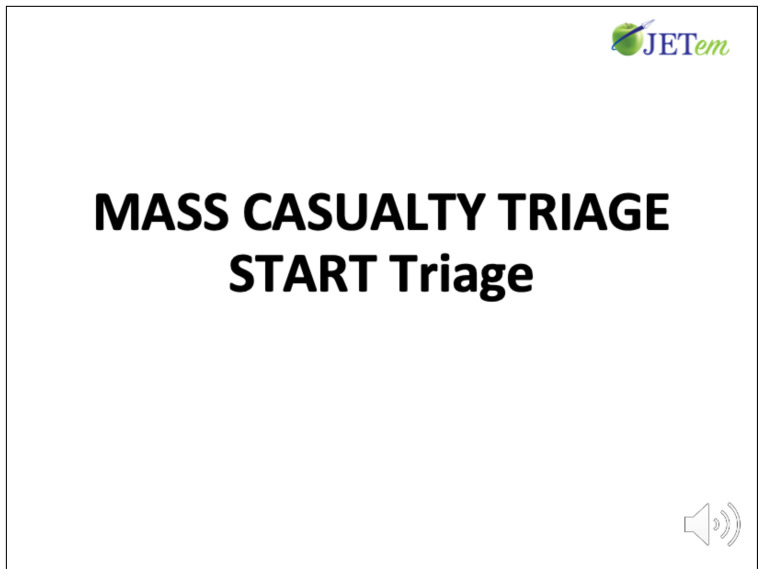
Video Link: https://youtu.be/UtfkiX99va0

**Figure f7-9-3-sg1:**
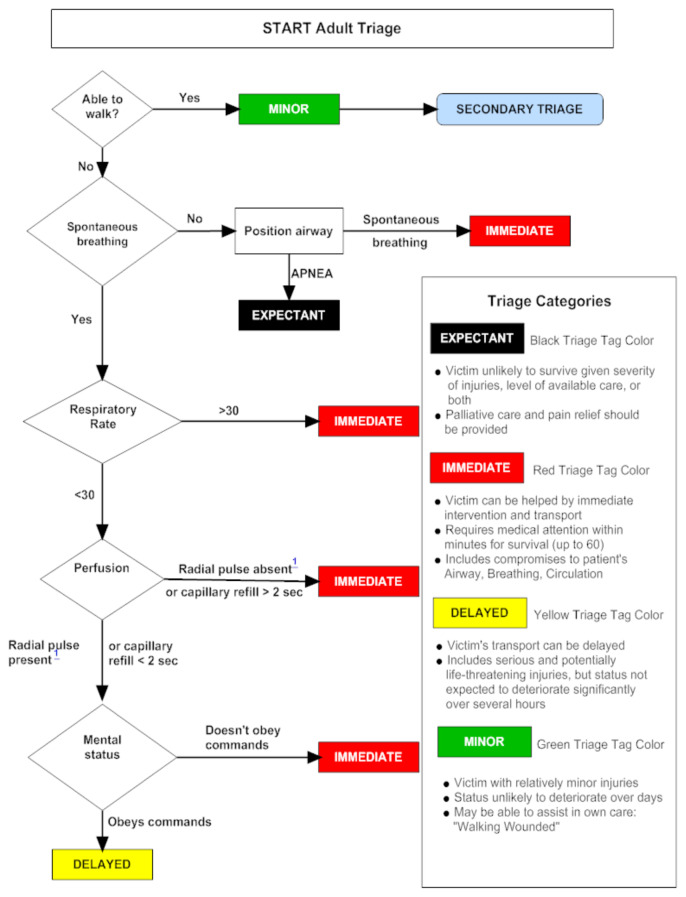
US Department of Health and Human Services. START Adult Triage Algorithm. In: Chemical Hazards Emergency Medical Management. https://chemm.hhs.gov/startadult.htm. Accessed April 10, 2024. Public Domain.

**Figure f8-9-3-sg1:**
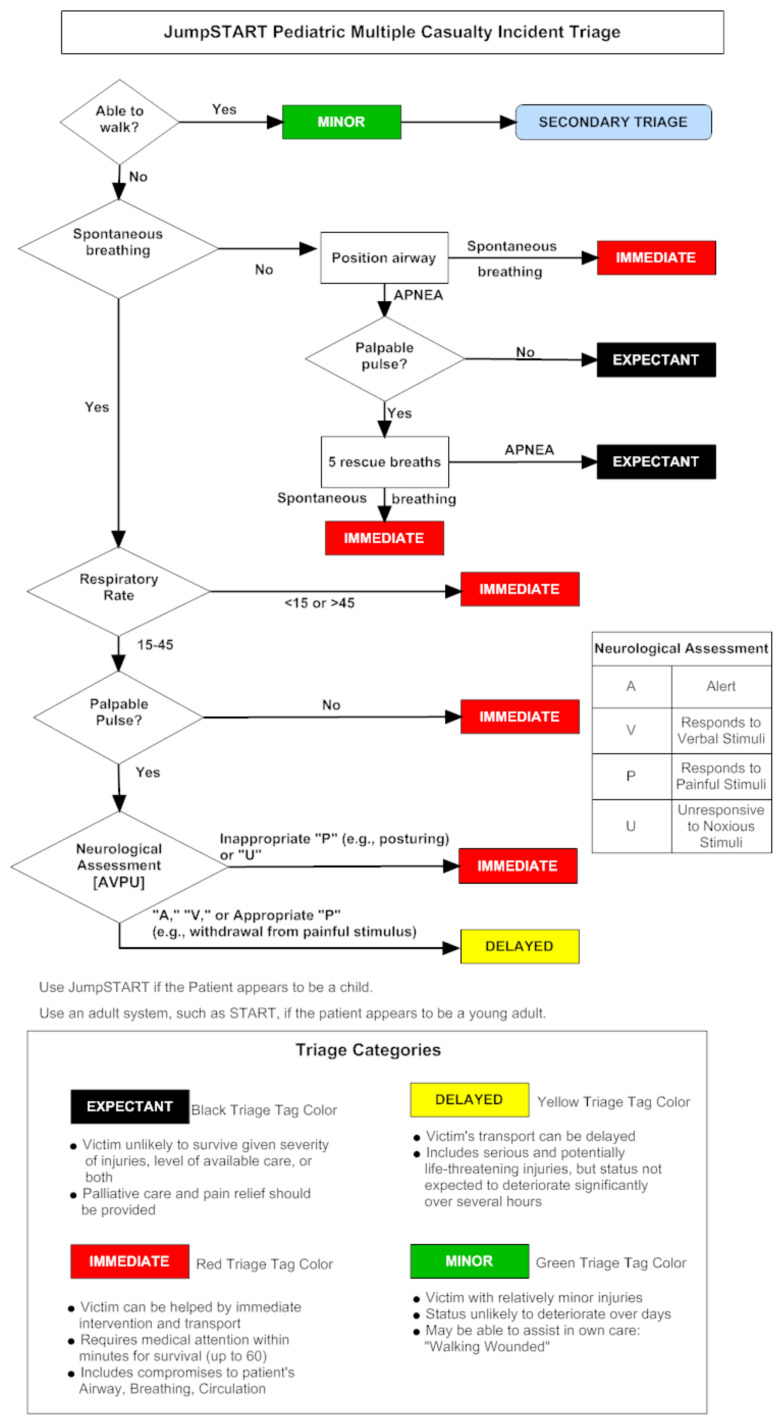
US Department of Health and Human Services. JumpSTART Pediatric Triage Algorithm. In: Chemical Hazards Emergency Medical Management. https://chemm.hhs.gov/startpediatric.htm. Accessed April 10, 2024. Public Domain.

**Figure f9-9-3-sg1:**
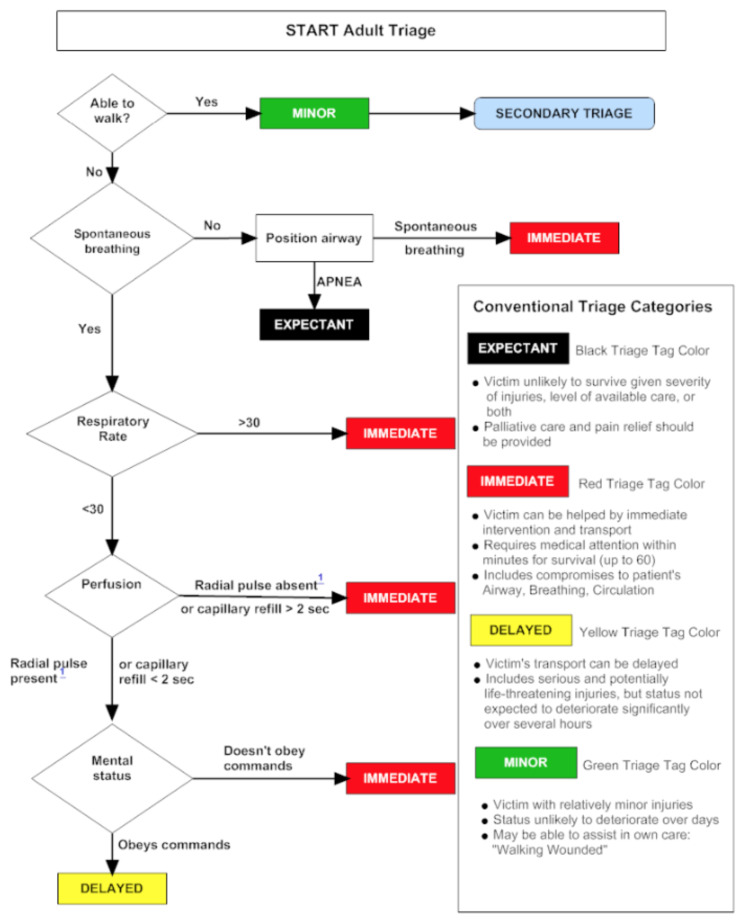
US Department of Health and Human Services. START Adult Triage Algorithm. In: Chemical Hazards Emergency Medical Management. https://chemm.hhs.gov/startadult.htm. Accessed April 10, 2024. Public Domain.

## SMALL GROUPS LEARNING MATERIALS

### Appendix A START Triage Pre-Test Questions and Answers

https://docs.google.com/forms/d/1cAMMjVVgSTN9pE0j0UxTxiT1LgPZBXzUIKP9D3flnM8/edit?usp=sharing_eip_m&ts=66a5bc89

***Note: To use the Google Forms, first “Make a Copy” so that you have a copy of the form for which you are the owner and can see the responses.**

1. What does START stand for?
  Select one:
  - Secure scene, Triage, Alert, Rapid Transport
  - Survey casualties, Treat patients, Assist evacuation, Request resources, Transport
  - Simple Triage and Rapid Treatment
  - Survey scene, Treatment, Airway, Re-assess, Transport
2. A 2 yo M at the scene of a subway explosion is brought to you by a frantic mother screaming, “He’s not breathing!” On rapid assessment, there is no spontaneous breathing after airway positioning. Using the Jump-START Triage Algorithm, what is the most appropriate next step?
  Select one:
  - Pulse check
  - 5 rescue breaths
  - Re-position airway
  - Move to next patient
3. A 25 yo F at the scene of a multi-car pile-up on the highway. She is ambulatory on-scene. She has a traumatic right upper extremity amputation at the elbow. You place a tourniquet with good hemostasis. Her RR is 24. Which START group does this patient belong to?
  Select one:
4. The same 25 yo F (with the RUE amputation), but now she is unable to ambulate. Her RR is 24. A&O x 3. What is the most appropriate START group for this patient now?
  Select one:
5. An 80’s M is a victim of a building collapse. The patient is unresponsive, without apparent injury. He is breathing spontaneously, RR 34. What is the most appropriate START triage category?
  Select one:
6. You are part of a rescue team responding to a landslide and come across a 30’s M in respiratory distress with blunt right chest injury. He is unable to ambulate, RR 30, A&O x 3. Which START Triage group is most appropriate?
  Select one:
7. The same patient as the previous question has now undergone a needle thoracostomy. His RR is now 20, A&O x 3. Which START group is most appropriate?
  Select one:
8. You are called to respond to a high-rise building fire. At the scene, you encounter a family of 3 including a 30’s M with traumatic left below elbow amputation. He has a tourniquet in place and is ambulatory with normal RR and normal mentation. The second is a 30’s F with obvious closed deformity to bilateral lower extremities and a RR of 22, normal cap refill, A&O x 3. The third is an infant who is not breathing. The infant begins to breathe after airway positioning. What is the most appropriate START triage category for each family member, in order?
  Select one:
  - , ,
  - , ,
  - , ,
  - , ,
9. You are assigned to the triage team at a bus accident with multiple victims. The first patient is a 3 yo child who is minimally responsive and apneic. You perform airway positioning. The child remains apneic. On further exam, the patient has a pulse. Per START Triage, what is next?
  Select one:
  - Give 5 rescue breaths
  - The patient is triaged as
  - The patient is triaged as
  - Clear foreign material from the airway.
10. You are on scene after a roll-over bus crash on a rural highway with approximately 60 passengers. You come across an 80’s F who is A&O x 3, RR 28 with left lower extremity injury. The extremity has visible deformity and a palpable distal pulse. Her capillary refill is < 2 seconds. What is the most appropriate START category for this patient?
  Select one:

#### START Triage Pre-Test Answers

1. c
2. b
3. d
4. b
5. b
6. b
7. d
8. a
9. b
10. d

### Appendix B START Triage Slides

[Fig f5-9-3-sg1]

### Appendix C START Triage Lecture Video

[Fig f6-9-3-sg1]

### Appendix D START Triage Algorithms

[Fig f7-9-3-sg1] [Fig f8-9-3-sg1]

### Appendix E START Triage Learning Scenarios for Online Modules

https://docs.google.com/forms/d/1B6V1YaDaVOy6tXNlyewswVrlFEkMsWhYI-NKoankGyY/edit?ts=66a5bc80

***Note: To use the Google Forms, first “Make a Copy” so that you have a copy of the form for which you are the owner and can see the responses.**

You are the first on scene to an accident of a school bus vs. a car. There are multiple injured patients, and you start to triage them. At the end of the scenario, you will be asked who you will decide to transport to the hospital first.

The first man you see looks to be in his 50’s walking around the scene, asking for help. As you approach he states, “My wife is in the car! Please help her!” You ask if he’s ok, which he responds in the affirmative. What is his triage category?

Select one:

- Minor
- Immediate
- Expectant
- Delayed

The correct answer is: Minor (Green) The patient is ambulatory, responding to verbal commands, and is oriented.

You walk up to the car and see a woman, maybe mid 40’s, head against the steering wheel, blood noted from the ear. You shake her with no response. You hear minimal breath sounds, RR 20’s, carotid pulse present HR>100. What is her triage category?

Select one:

- Minor
- Immediate
- Expectant
- Delayed

The correct answer is: Immediate (Red) This patient does not have appropriate mental status

What Interventions should be performed to more accurately triage this patient?

Select one:

- Apply a tourniquet
- Open airway
- No further interventions necessary
- Check cap refill

The correct answer is: No further intervention is necessary The patient passes R-P of R-P-M; however, she does not have mental status. The patient is already breathing with a RR < 30. Her radial pulse is present. The patient does not obey commands. Based upon the question stem, she is not a candidate for the 4 field interventions: Airway maneuvers, needle decompression, tourniquet application, or an antidote.

You enter the bus and note the driver is screaming in pain with visible deformity to the left arm with glass shards protruding from the skin. Patient repeatedly states it hurts. Radial pulses are intact, RR 28, HR 118. You ask him if he can move, and he states he can’t move his arm but is able to move other extremities. What triage category is this patient?

Select one:

- Minor
- Immediate
- Delayed
- Expectant

The correct answer is: Delayed (Yellow) This patient has a single limb injury. ABCs are clearly intact as he is noisy and moving ¾ limbs. The question does not indicate evidence of severe arterial extravasation at this time; therefore, the patient does not need a tourniquet.

You note a roughly 30’s male is on the bus floor. He wakes up when you shake him. He’s notably tachypneic, RR 32, and bleeding down his leg with his foot stuck between the bus seat. Radial and femoral pulses palpable.

Which of the following is the most appropriate triage category?

Select one:

- Minor
- Delayed
- Immediate
- Expectant

The correct answer is: Immediate (Red) When you assess R-P-M, the patient’s RR is > 30 and has Altered Mental Status (AMS), placing him in the Immediate category.

Please go to part 2 of this question.

Part 2: What other interventions should be done before moving on to the next patient?

Select one:

- Check capillary refill
- Apply a tourniquet above the site of bleeding
- Open airway
- Needle decompression to chest
- No further intervention necessary

The correct answer is: Apply a tourniquet above the site of bleeding For those patients classified as Immediate/Red, brief life-saving interventions should be performed prior to moving on to the next patient. These consist of:

1. Open the airway
2. Tourniquet application
3. Needle decompression
4. Antidote administration

Please go to Part 3 of this patient.

Part 3: Patient #4 (30’sM with a pinned leg, tachypnea and AMS) Continued... After intervention, he’s talking to you, screaming, in notable pain. He’s vitally stable, demanding transport to the hospital. Femoral pulses remain intact bilaterally. What is his final triage category?

Select one:

- Minor
- Delayed
- Immediate
- Expectant

The correct answer is: Delayed (Yellow) Life-saving intervention was indicated in this patient (tourniquet placement) and resulted in triage category improvement to Delayed (Yellow). Remember that triage categorization is not made until the R-P-M assessment and life-saving interventions have both been performed.

You have now evaluated every patient on scene. EMS crews are arriving. Please put the patients in transportation order, from first to transport to last to transport.

____ Male, ambulatory on scene
____ Male, pinned leg, tourniquet in place
____ Woman, driver of car, unresponsive
____ Male, driver of bus, glass in arm

The correct answer is: 4, 2, 1, 3

1: [woman, driver of car, unresponsive]
2: [male, pinned leg, tourniquet in place]
3: [male, driver of bus, glass in arm.]
4: [male, ambulatory on scene]

First to transport is our Red tag, the woman who was the driver of the car. She is unresponsive with abnormal vital signs. Next, we have two Yellow tags: 1) the bus driver with glass in his arm who is very noisy and yelling. 2) the bus passenger with the pinned leg who required a tourniquet to be placed. The passenger goes first because he was initially going to be a Red tag; however, application of a tourniquet improved his clinical condition to Yellow. The bus driver is not requiring life-saving intervention in the field and therefore should be transported third. Last to be transported is our green tag patient, the male passenger from the car who is the first patient we encounter when we arrive on scene.

### Appendix F Multiple Casualty Incident (MCI) Simulation

**Scenario:** A tropical storm hit the Bronx resulting in the Leon chemical factory being struck by lightning. There were multiple explosions and leakage of chemicals from the factory. FDNY calls stating multiple casualties and additional ambulances are on route to arrive in 10 minutes. We need you to define their injuries and rapidly triage these patients.

**Brief narrative description of cases/scenario:**

This simulated MCI requires learners to apply rapid triage, using the START triage algorithm and under time constraint, to six simulated patients. The scene is either in the field or in the ambulance bay of the receiving hospital, and therefore resources are extremely limited.

**Learning Objectives:**

By the end of this activity, learners will be able to:

1. Reliably triage patients into treatment categories based upon START Triage.
2. Demonstrate accurate application of the START Triage algorithm under stress.
3. Demonstrate appropriate use of acute life-saving interventions as dictated by the START triage algorithm.
4. Identify and treat tension pneumothorax.
5. Identify patients requiring decontamination from presumed chemical exposure prior to emergency treatment.
6. Demonstrate appropriate use of CPR in an MCI for a high voltage electrocution.

**Critical Actions:**

1. Identify the need to perform acute life-saving interventions before moving to next patient.
2. Perform needle decompression on tension pneumothorax.
3. Send chemical exposure patients for decontamination before entry to the emergency department.
4. Perform CPR on a high voltage electricity or lightning strike victim prior to declaring the patient dead.
5. Stay within the time limit for each patient scenario.
6. Appropriately triage each patient

**Learner preparation/prework:**

All learners will have completed the pretest and online learning module before attending the MCI scenario.

**Setup/Materials** ***:***

- 5 simulated patients.
- Instructor to also act as timekeeper
- An austere area to run cases or a simulation center stripped of most equipment.
- Basic triage materials –
  ○ Stethoscope
  ○ IV catheters/antidote
  ○ Tourniquets
  ○ Bandaging supplies
- Patients can be holding pieces of paper with vital signs on them to hand to learners at appropriate times during the case.

**Patient Scenarios for Simulated MCI:**

**Patient #1**

Scenario overview: Patient was thrown by an explosion while exiting the chemical plant. The patient has loud tinnitus and visible bruising to skin over right chest. The patient has tachycardia and tachypnea and is unable to answer questions due to hearing loss. The patient is found to have a Right side tension pneumothorax, and appropriate intervention would be a needle decompression.

Scenario Flow:[Table t2-9-3-sg1]

**Table t2-9-3-sg1:**

Intervention/Timepoint	Change in Case	Additional Information
Vital Signs	HR 126, RR 34	Patient with respiratory distress, “Red tag” category at this point.
Doctor asks a question	Patient response: “What? What?”	
Doctor asks again	Patient response: “I have ringing in my ears! I can’t hear!”	Presumed ruptured tympanic membranes (TMs) from blast injury.
Examination	If lungs auscultated →	Absent Breath sounds on right. Bruising to right side of chest.
Needle decompression	If performed →	Pt. reports feeling better. VS: 146/82, HR 106, RR 28, O2 96%. “Yellow tag” category.
	If not performed →	Pt. remains in distress, no decompensation. VS unchanged. “Red tag” category.
End scenario		

**Patient #2:**

Scenario Overview: Patient was hit by flying metal, which is now sticking out of the abdomen. Vitals unstable. Patient is lying on the ground with obvious metal in place. Patient moaning in pain, answers simple questions. The patient only remembers the explosion and feeling like he was hit by something. Learners should brace the metal with bandaging and instruct the patient or an observer to apply pressure to bleeding.

Scenario flow:[Table t3-9-3-sg1]

**Table t3-9-3-sg1:**

Intervention/Timepoint	Change in Case	Additional Information
VS	HR 120, RR 30	Patient unstable, “Red tag” at this point, can decompensate quickly.
Doctor asks a question	Patient response: “Uhhh--all I remember is sound of explosion and woke up on the floor. My belly hurts.”	Trainee should note impaled object with active bleeding.
Hemorrhage Control and stabilization of impaled object	If performed →	Bleeding controlled. VS: 105/68, HR 112, RR 30, O2 96%. “Red tag” category.
	If not performed ->	Patient becomes unresponsive and dies. “Black tag” category.
End scenario		

**Patient #3:**

Scenario Overview: Patient is unresponsive with obvious large gaping head wound, no vital signs.

Scenario Flow:[Table t4-9-3-sg1]

**Table t4-9-3-sg1:**

Intervention/Timepoint	Change in Case	Additional Information
VS	HR N/A, RR N/A	Pt. w/unresponsive, gaping head injury, black tag.
Doctor asks a question	Patient response: none	
Doctor asks again	Patient response: none	Catastrophic head injury, category “deceased/expectant.”
If learners attempt CPR	Patient response: none	No return of spontaneous circulation (ROSC). Learners will be prompted to move on to next patient by timekeeper after 30 seconds.
End scenario		

**Patient #4:**

Scenario Overview: The patient is a chemical factory worker and was working in the chemical storage area at the time of the explosion. The patient is AOx3, speaking few word sentences due to pain and dyspnea. There are secretions and mucus with cough and difficulty breathing and diaphoresis. The patient says symptoms began when a chemical barrel ruptured.

Scenario Flow:[Table t5-9-3-sg1]

**Table t5-9-3-sg1:**

Intervention/timepoint	Change in Case	Additional Information
VS	HR 48, RR 32	Pt. w/resp. distress. “Red tag” category at this point.
Doctor asks a question	Patient response: “I can’t breathe.” The chemicals spilled, significant coughing, speaking partial sentences	Presumed chemical exposure.
Doctor asks about other symptoms	Patient response: “I am very sweaty, my lungs hurt!”	
Examination	If lungs auscultated —>	Tachypneic, scant rales b/l, mild increase in work of breathing, no stridor.
Examination	If skin examined →	Diaphoresis to visible skin.
Doctor	Diagnoses cholinergic toxidrome	
	Patient flagged for decontamination	Pt. remains in distress. VS unchanged. “Red tag” category.
End scenario		

**Patient #5:**

Scenario Overview: The patient is unconscious with no signs of life; however, there is evidence to suggest that he has had a cardiac arrest due to a high voltage electrical injury. His shirt identifies him as an electrician, and his skin has been moulaged to show a ferning pattern consistent with high-voltage electrical exposure. In this scenario, CPR is indicated prior to moving to the next victim.

Scenario Flow:[Table t6-9-3-sg1]

**Table t6-9-3-sg1:**

Intervention/Timepoint	Change in Case	Additional Information
VS	HR 0, RR 0	Pt. is next to an electricity box, if available.
Doctor action:	Perform CPR	This patient is in cardiac arrest due to electrical injury.
	In less than 1 cycle of CPR, patient has ROSC and wakes up.	Stop CPR, assess patient.
VS	HR 110, RR 22	
Doctor asks patient a question:	Patient reports, “I was going to shut off the main power after the explosion and I got electrocuted.”	Patient triaged as “Red tag,” considering he just had a cardiac arrest.
End scenario		

**Patient #6:**

Scenario Overview: The patient is found sitting on the side of a structure (ie: fence or building) with normal vitals, physical exam of patient without any findings. Patient talking and stable.

Scenario Flow:[Table t7-9-3-sg1]

**Table t7-9-3-sg1:**

Intervention/timepoint	Change in Case	Additional Information
VS	HR 84, RR 18,	Pt. is next to the structure, sitting up.
Doctor action:	Approach patient, introduce self and access	Pt. states he/she is having and was thrown, otherwise feels fine.
	Patient exam, asking patient to stand up and walk	Patient is able to follow all commands.
VS	HR 86, RR 18	
Doctor:		Patient triaged as “Green Tag,” vitals stable, and able to follow simple commands.
End scenario		

**Ideal Scenario Flow:**

Ideally, this scenario is run under a time constraint with 60 seconds given for triage and intervention on each patient. Learners can work individually or in teams of two-three residents, moving from patient-to-patient to triage. An instructor will act as timekeeper, signaling learners when to move to the next patient.

Learners will complete triage of all six patients without discussion with others. After all of the learners in the group have completed the six scenarios, the group gathers to go over the scenarios and debrief with analysis of application of the START triage pathway to each patient.

**Debriefing materials and “answers” to patient triage scenarios:**

**“Answers”**

Patient 1: Yellow tag. Needle decompression is a critical action.
Patient 2: Red tag. Hemorrhage control is a critical action.
Patient 3: Black tag. The patient is dead.
Patient 4: Red tag. Flagging the patient for decontamination is a critical action.
Patient 5: Red tag. Performing CPR on a high-voltage electrical cardiac arrest is the one exception to “no CPR” during START triage.
Patient 6: Green tag. The patient has normal vitals and no exam evidence requiring critical action.

**Sample questions for debriefing:**

General Discussion for Trainees:

- Can you summarize this case scenario?
- How do you think your team performed on the scenario?
- Was this environment and situation comfortable for you, or difficult? Why?
- What do you believe went well during the simulation?
- What do you believe did not go well? Anything you would have changed?

Medical knowledge:

What are the “3 vital signs” of START Triage?

- “R-P-M,” Respiratory Rate, Heart Rate, Mental Status.

What are the three critical actions mandated by START Triage before moving onto the next patient?

1. Open Airway
2. Hemorrhage Control
3. Needle Decompression of tension pneumothorax

Why should the electrical injury patient (Case 5) receive CPR when no other patients do?

- Electrocution injury/lightning strike patients are the exception to the rule of “no CPR” because they have a high likelihood of rapid ROSC.

Case 4 has a clear toxidrome due to chemical exposure. What is going to be a critical action for this patient before healthcare can proceed?

- Patients with chemical exposure need to undergo decontamination before entering the healthcare setting to stop the continued toxic exposure of the patient as well as to protect caregivers.

**Triage algorithm:** [Fig f9-9-3-sg1]

### Appendix G START Triage Pocket Card

Print double-sided. Can be made smaller as an ID Badge Tag.[Fig f10-9-3-sg1]

Adapted from: Thoma B. Tiny Tips: START Protocol for Mass Casualty Triage. Published May 13, 2021. Accessed April 10, 2024. In: CanadiEM https://canadiem.org/tiny-tips-start-protocol-for-mass-casualty-triage/

### Appendix H 3-months Delayed Post-Test and Answers

https://docs.google.com/forms/d/1nv6wKCLZUWPc2rYJs9PN7c8eg8_D9-rR9cFnYj_SNH4/edit?ts=66a5bc78#settings

***Note: To use the Google Forms, first “Make a Copy” so that you have a copy of the form for which you are the owner and can see the responses.**

Reassessment Questions:

1. What does START stand for?
  Select one:
  - Survey scene, Treatments, Airway, Reassess, Transport
  - Simple Triage and Rapid Treatment
  - Secure Scene, Triage, Alert, Rapid Transport
  - Survey Casualties, Treat patients, Assist Evacuation, Request Resources, Transport
2. A 2 yo M at the scene of a subway explosion is brought to you by a frantic mother screaming, “He’s not breathing!” On rapid assessment, there is no spontaneous breathing after airway positioning. Using the Jump-START Triage Algorithm, what is the most appropriate next step?
  Select one:
  - 5 rescue breaths
  - Pulse check
  - Re-position airway
  - Move to the next patient
3. 25yo F at the scene of multi-car pile up on the highway. She is ambulatory on-scene. She has a traumatic right upper extremity amputation at the elbow. You place a tourniquet with good hemostasis. Her RR is 24. Which START group does this patient most appropriately belong?
4. Same 25yo F with RUE amputation, but now she is unable to ambulate. RR 24, AOx3. What is the most appropriate START group for this patient now?
5. 80’s M At the scene of building collapse after an earthquake. The patient is unresponsive, without apparent injury. He is spontaneously breathing, RR 34. What is the most appropriate START category?
6. You are part of a rescue team responding to a landslide and come across a 30’s M in respiratory distress with blunt right chest injury. He is unable to ambulate, RR 30, AOx3. Which START group is most appropriate?
7. Same patient as above, after needle thoracostomy has RR 20, AOx3. Which START group may this patient be appropriately placed in?
8. You are called to respond to a high-rise building fire after suspected terrorist attack. At the scene you encounter a family of 3: A 30’s M with traumatic left below elbow amputation with tourniquet in place. He is ambulatory with normal RR and mentation. A 30’s F with obvious closed deformity to bilateral lower extremities and RR 22, normal capillary refill, AOx3. An infant who is not breathing. The infant begins to breathe after airway positioning. What is the most appropriate START grouping from the order of presentation above?
  - , ,
  - , ,
  - , ,
  - , ,
9. You are assigned to Triage Team at a bus accident with over 20 victims. First patient is a 3yo child who is minimally responsive, you perform airway positioning, and the child remains apneic. On further exam the pt. has a pulse. What is the next step?
  - The pt is triaged as
  - Give 5 rescue breaths
  - The patient is triaged as
  - Clear foreign material from airway
10. You are on scene after a roll-over bus crash on a rural highway with approximately 60 passengers. You come across an 80’s F who is AOx3, RR 28 with a left lower extremity injury. The extremity has visible deformity and palpable distal pulse. What is the most appropriate START category for this patient?

#### 3-months Delayed Post-Test Answers

1. B
2. B
3. A
4. D
5. A
6. D
7. D
8. B
9. B
10. D
