# Supplementary material for: Utilization of an Asynchronous Online Learning Module Followed by Simulated Scenario to Train Emergency Medicine Residents in Mass-Casualty Triage
Source: J Educ Teach Emerg Med. 2024 Jul 31;9(3):SG1–SG35. doi: 10.21980/J89S7Z (PMC11312877; doi:10.21980/J89S7Z)
Supplement: Supplementary file 1 [file 9-3-SG1-AppendixB.pptx]

## Slide 1
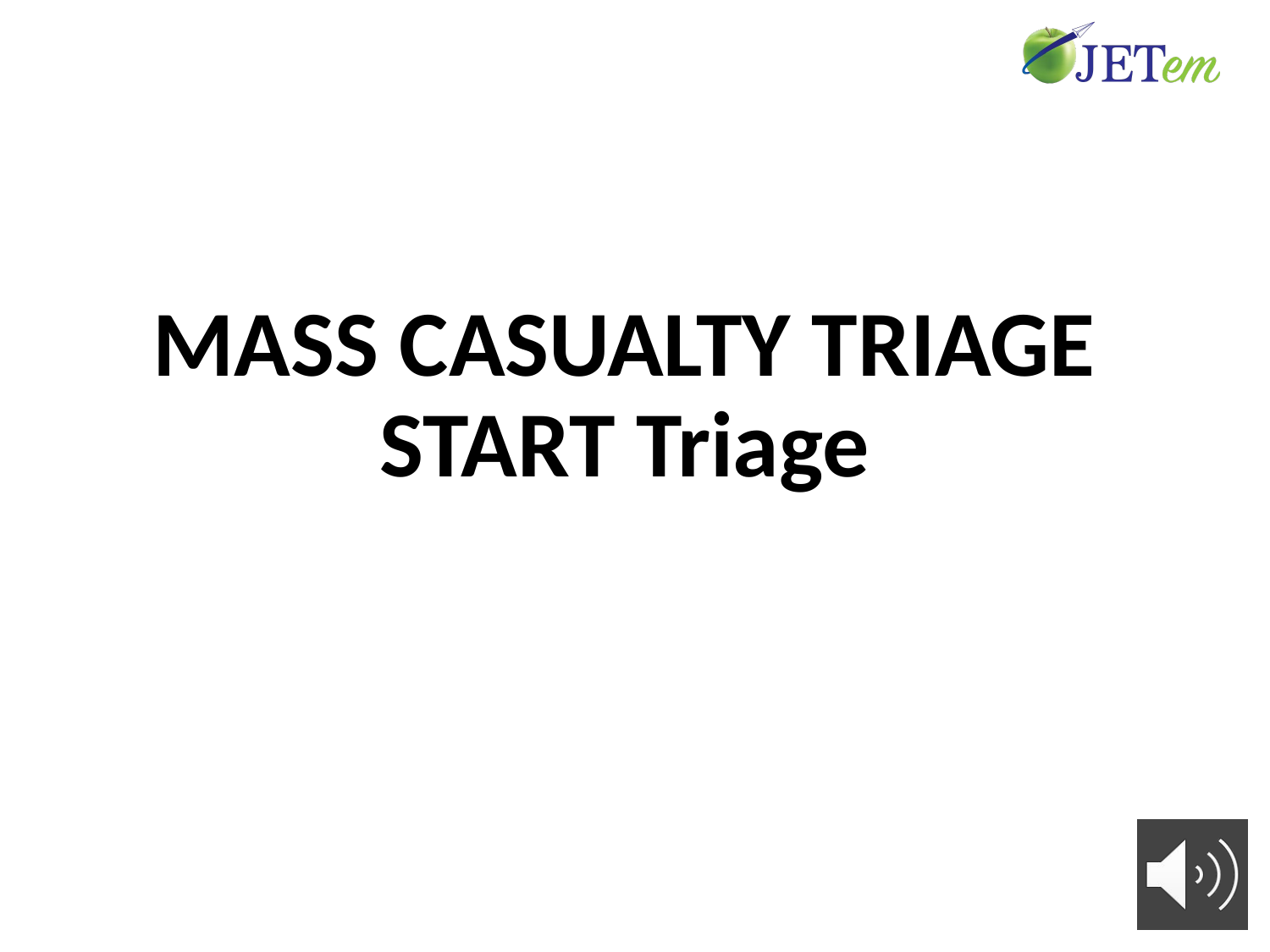

# MASS CASUALTY TRIAGE START Triage

## Slide 2
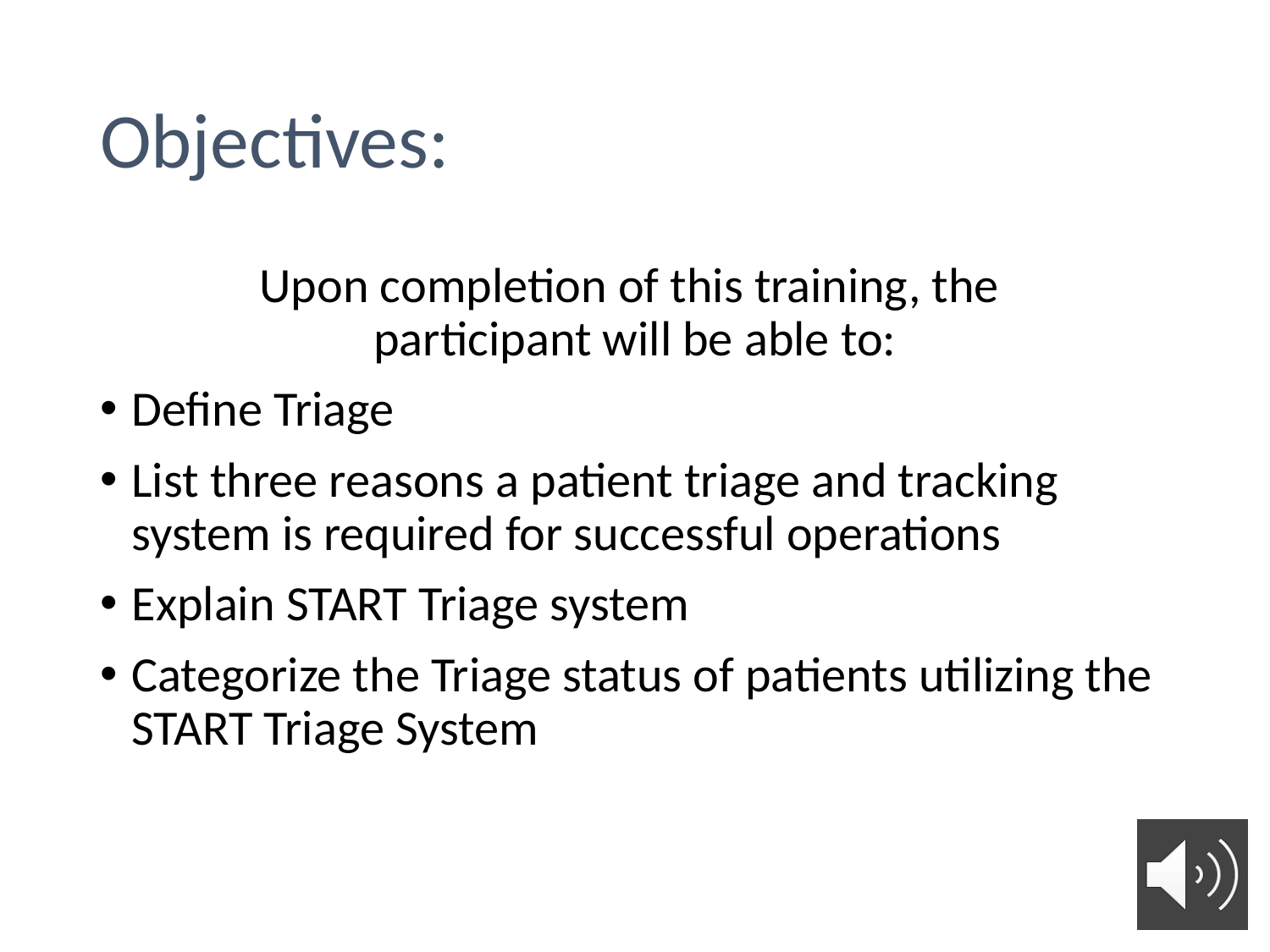

# Objectives:
Upon completion of this training, the
participant will be able to:
Define Triage
List three reasons a patient triage and tracking system is required for successful operations
Explain START Triage system
Categorize the Triage status of patients utilizing the START Triage System

## Slide 3
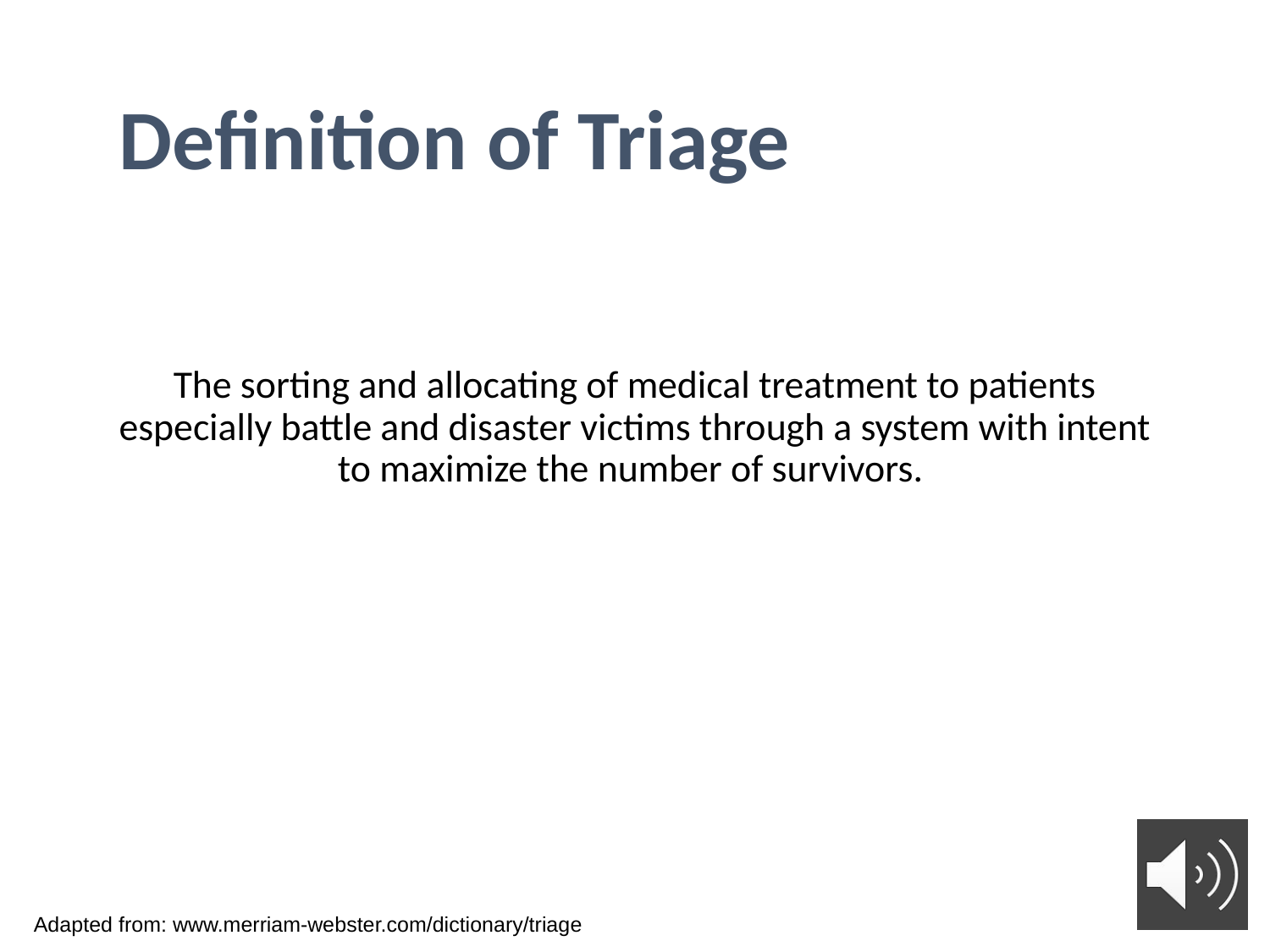

# Definition of Triage
The sorting and allocating of medical treatment to patients especially battle and disaster victims through a system with intent to maximize the number of survivors.
Adapted from: www.merriam-webster.com/dictionary/triage

## Slide 4
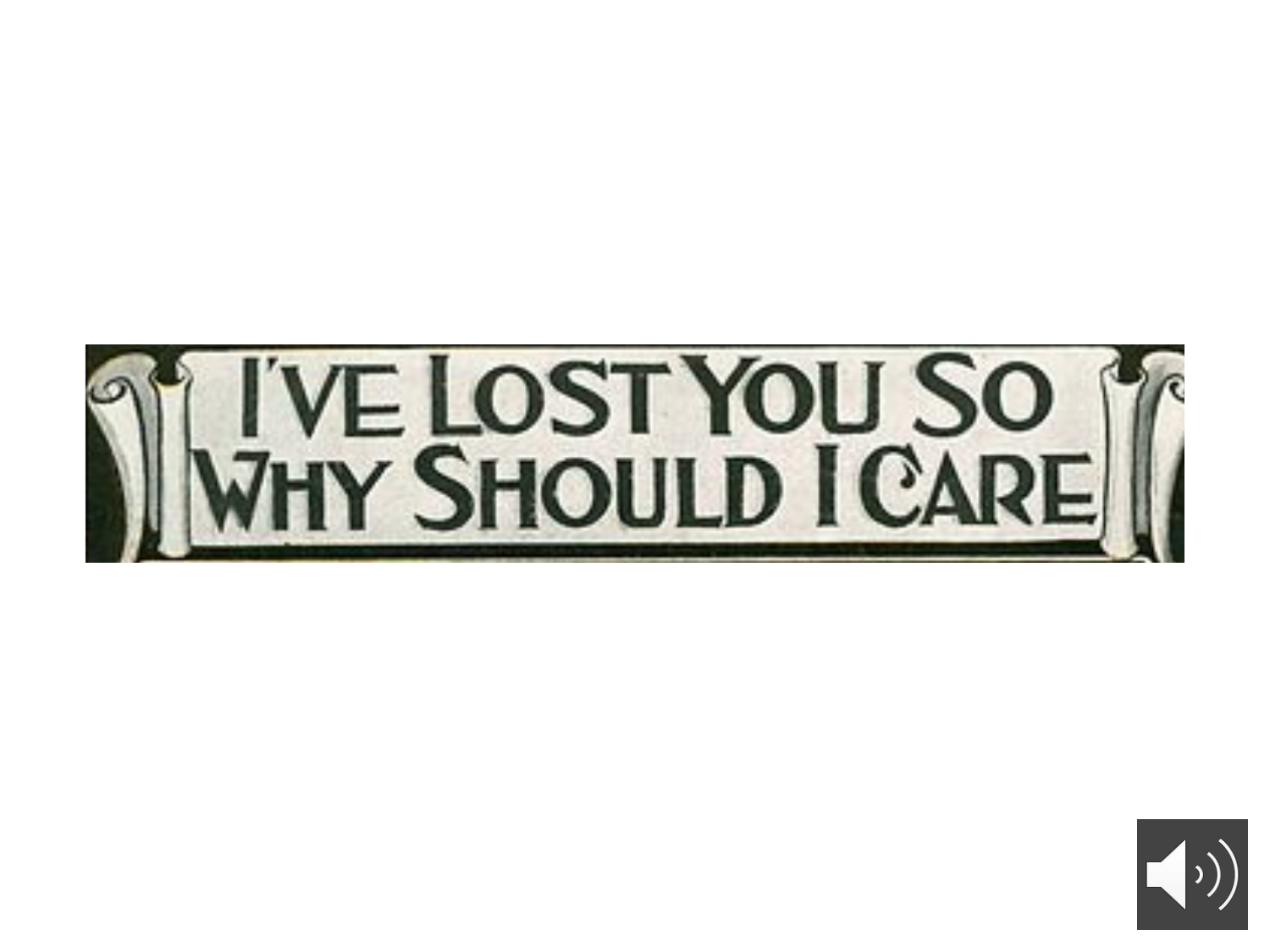

## Slide 5
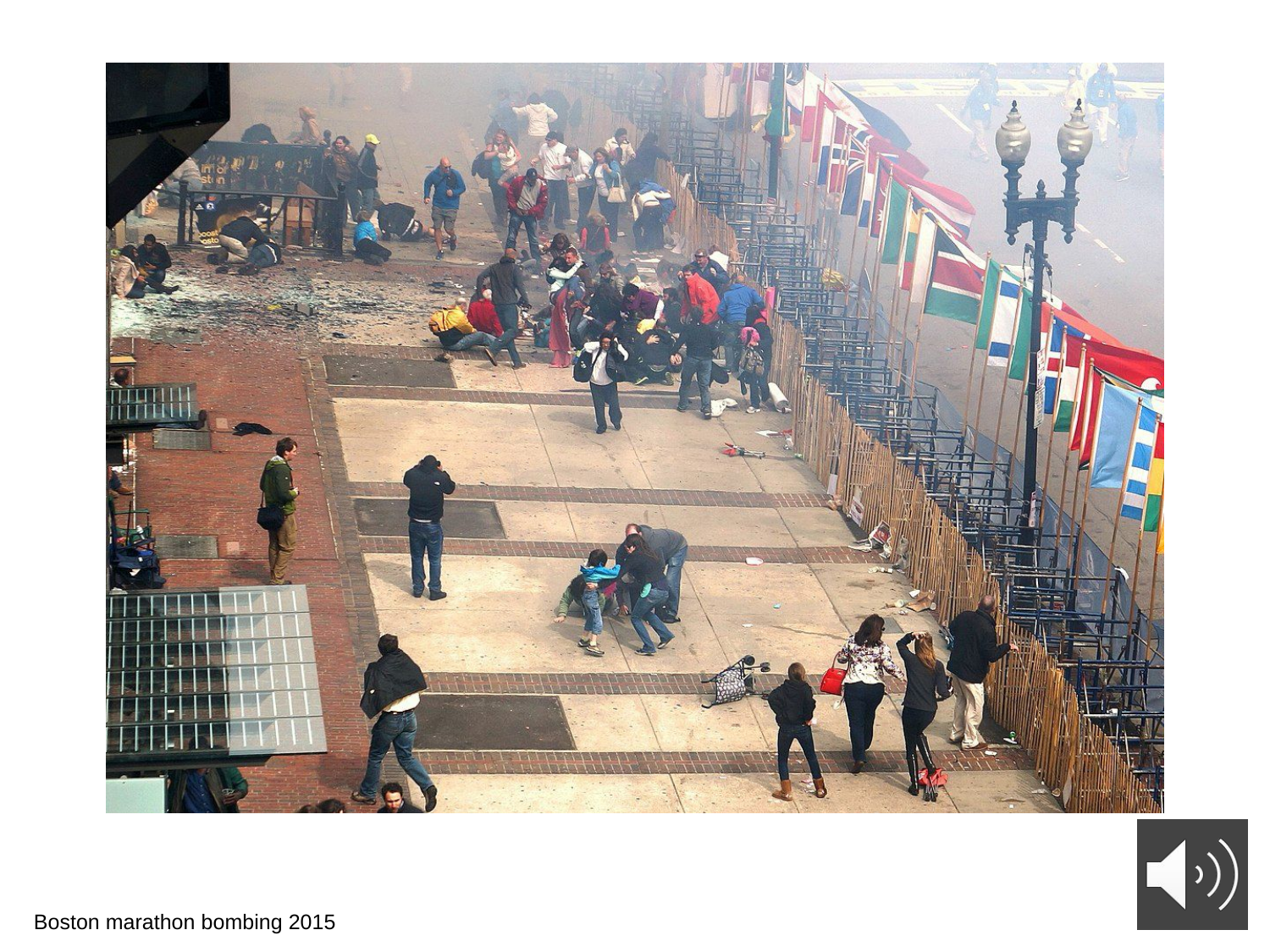

Boston marathon bombing 2015

## Slide 6
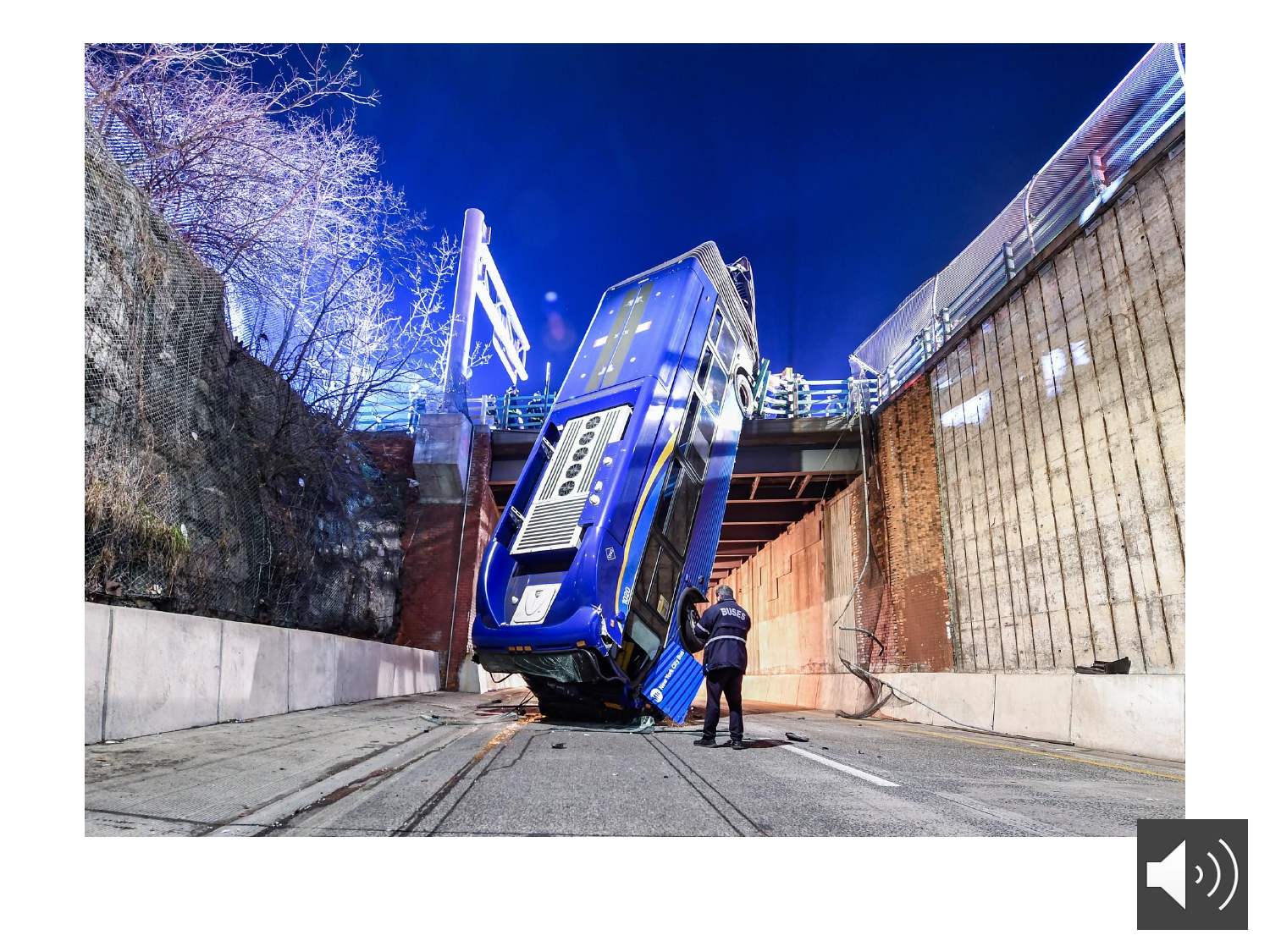

## Slide 7
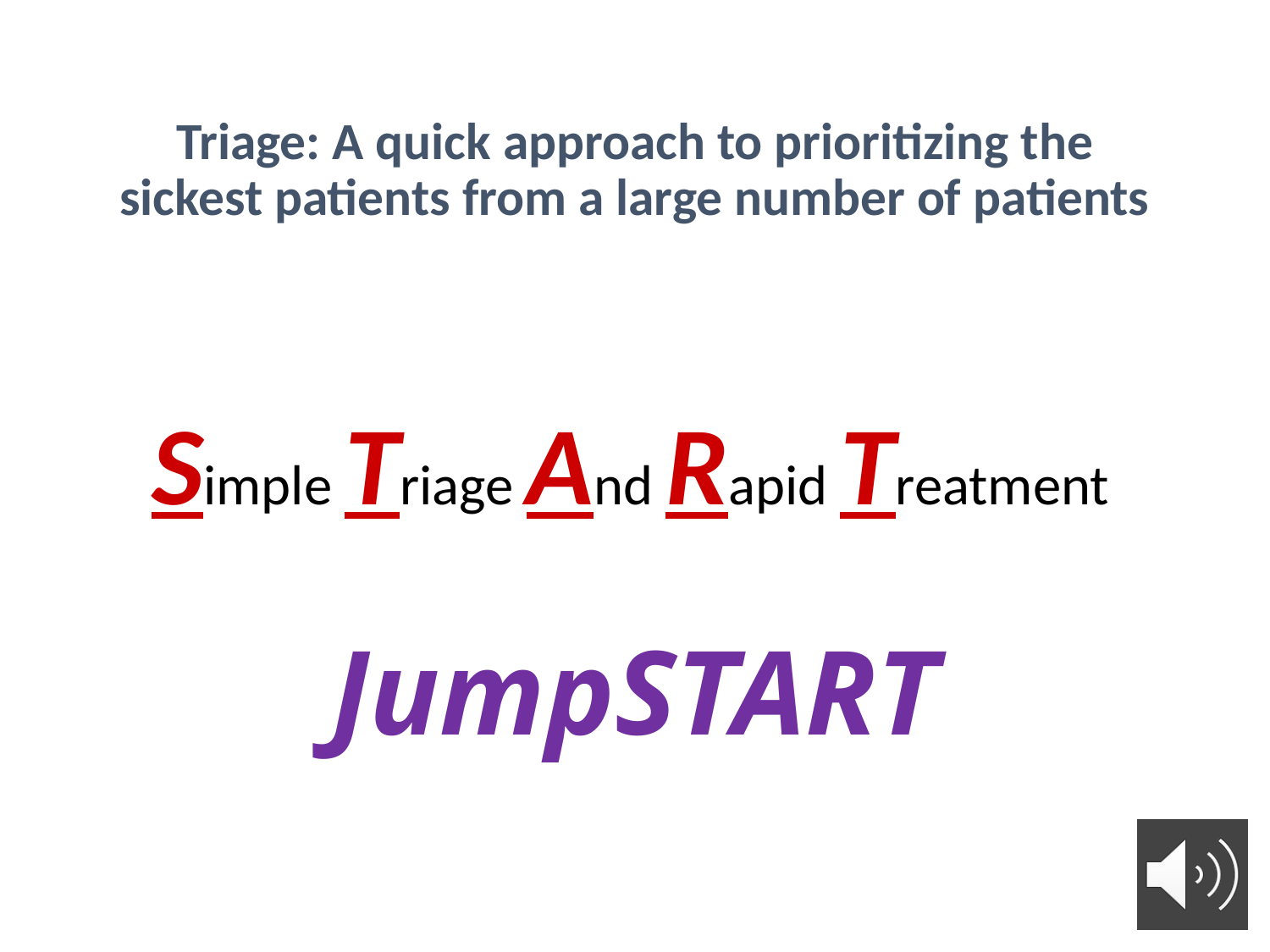

# Triage: A quick approach to prioritizing the sickest patients from a large number of patients
Simple Triage And Rapid Treatment
JumpSTART

## Slide 8
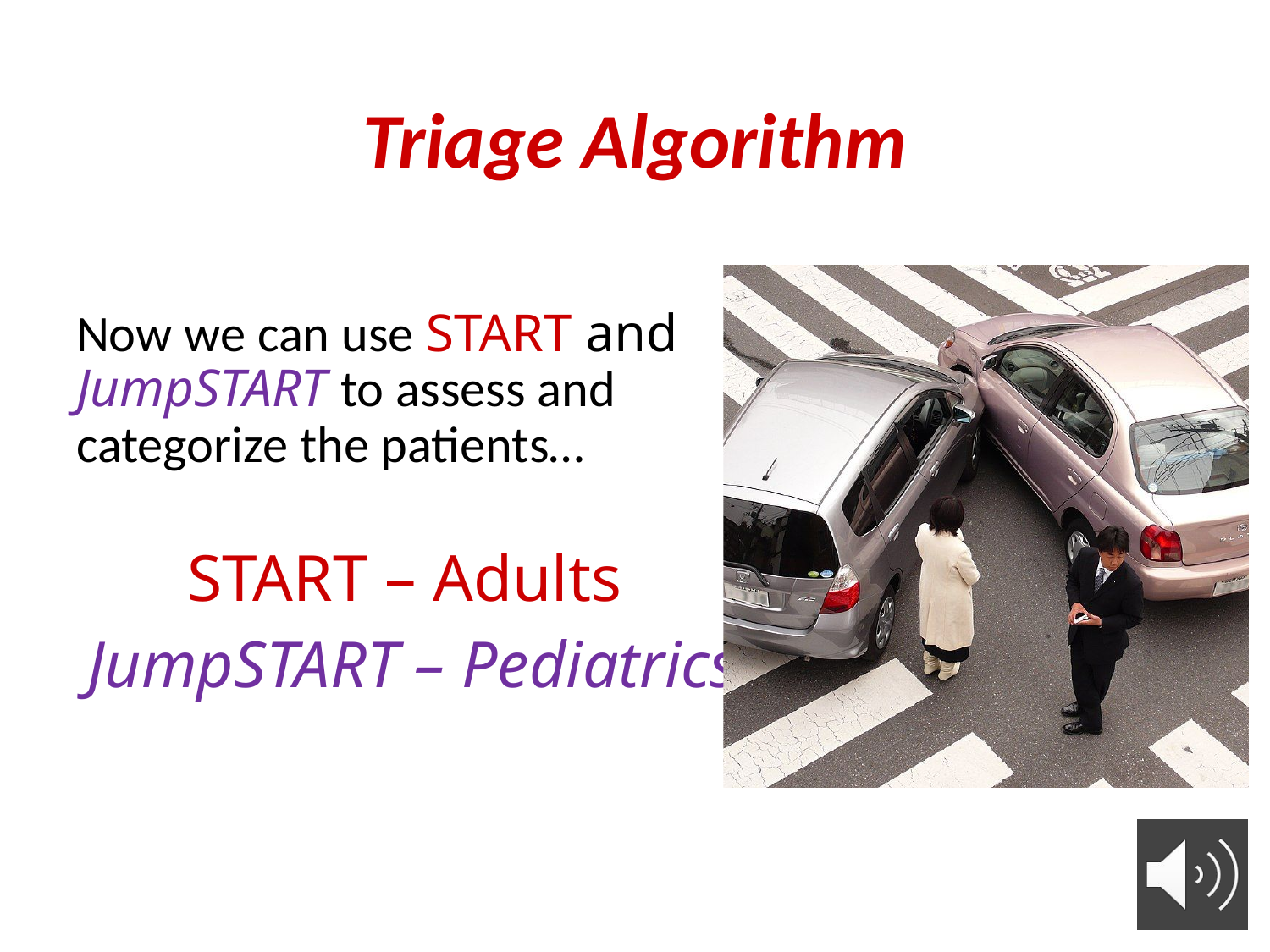

# Triage Algorithm
Now we can use START and JumpSTART to assess and categorize the patients…
START – Adults
JumpSTART – Pediatrics

## Slide 9
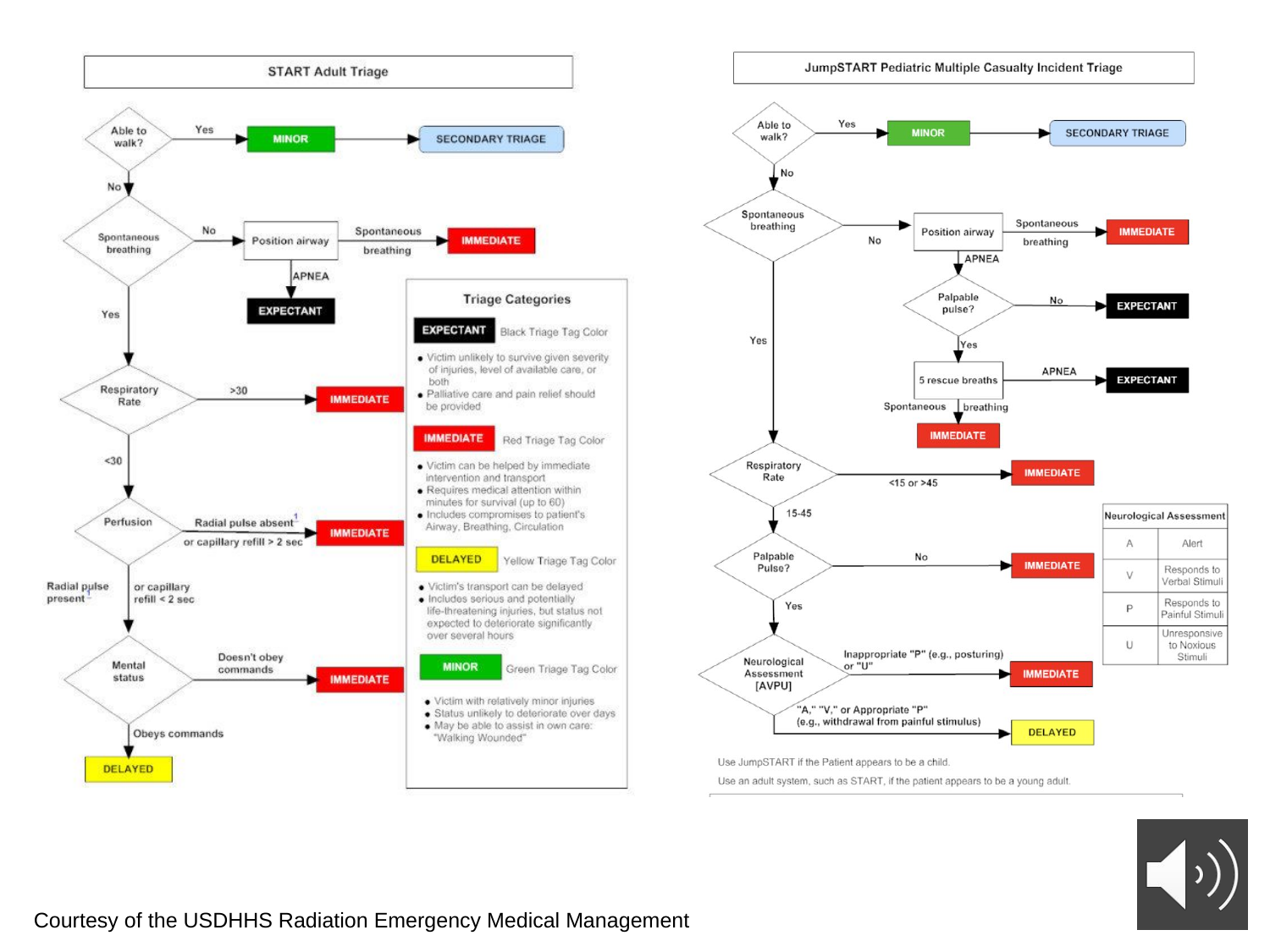

Courtesy of the USDHHS Radiation Emergency Medical Management

## Slide 10
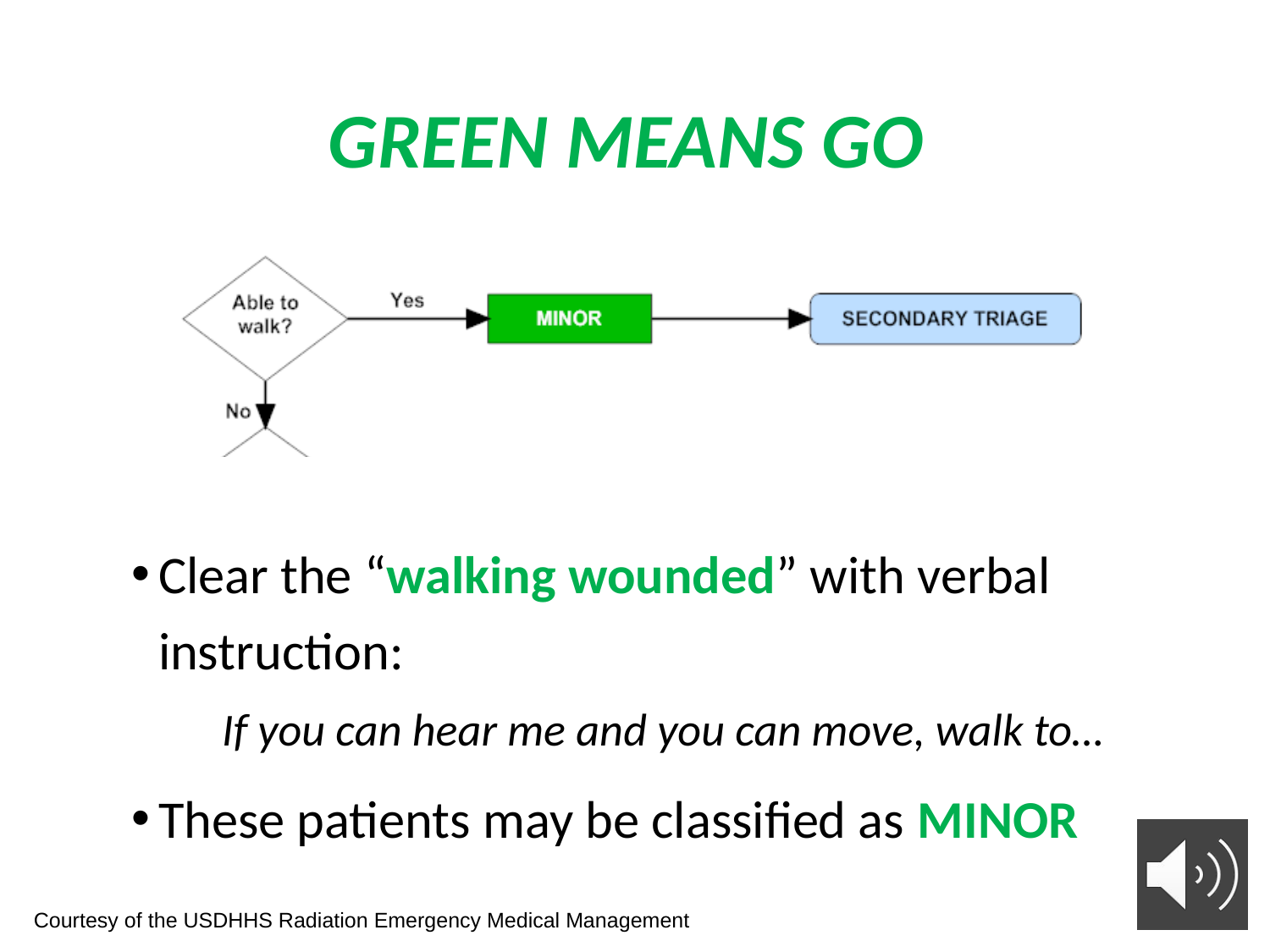

# GREEN MEANS GO
Clear the “walking wounded” with verbal instruction:
If you can hear me and you can move, walk to…
These patients may be classified as MINOR
Courtesy of the USDHHS Radiation Emergency Medical Management

## Slide 11
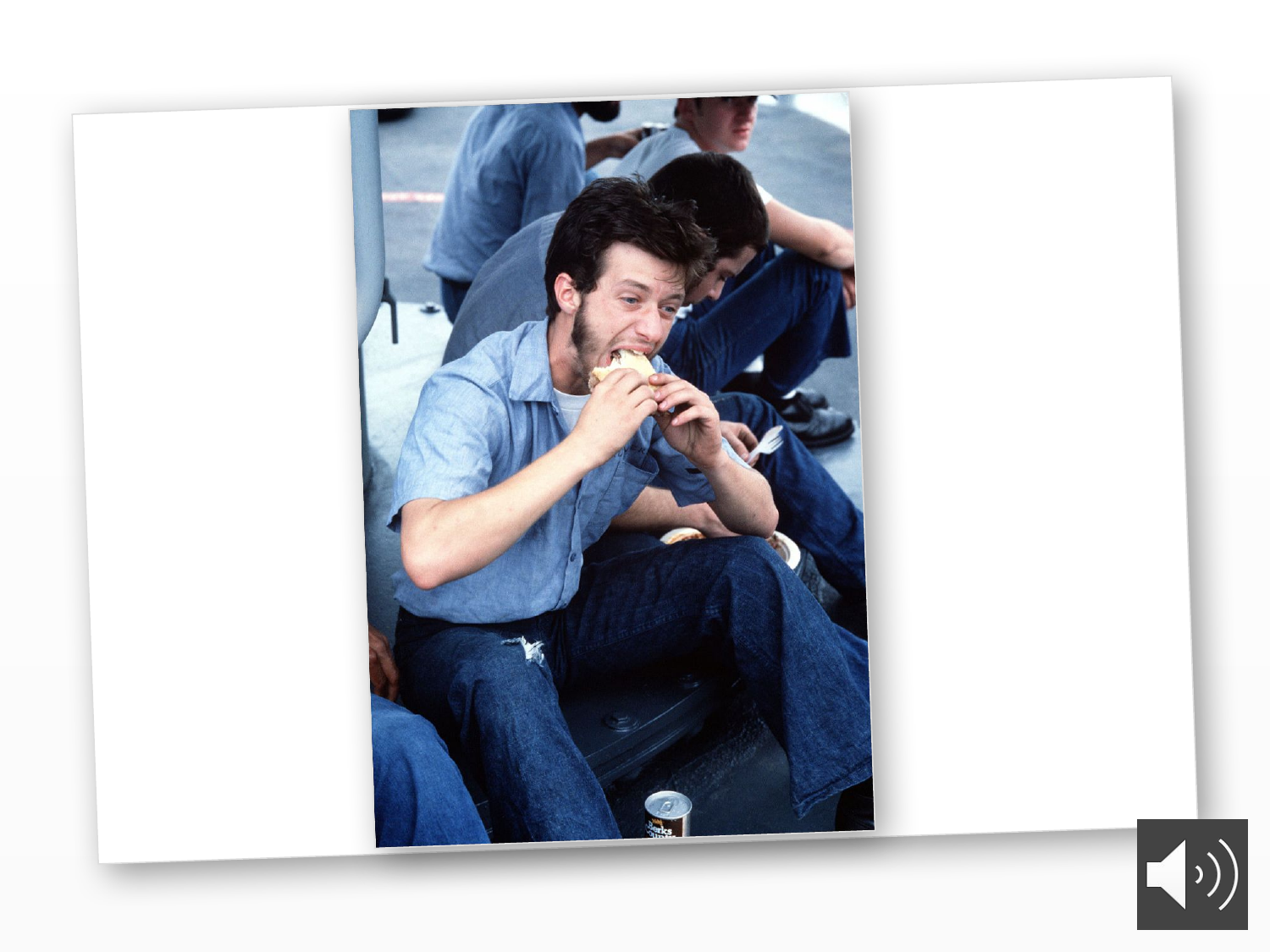

## Slide 12
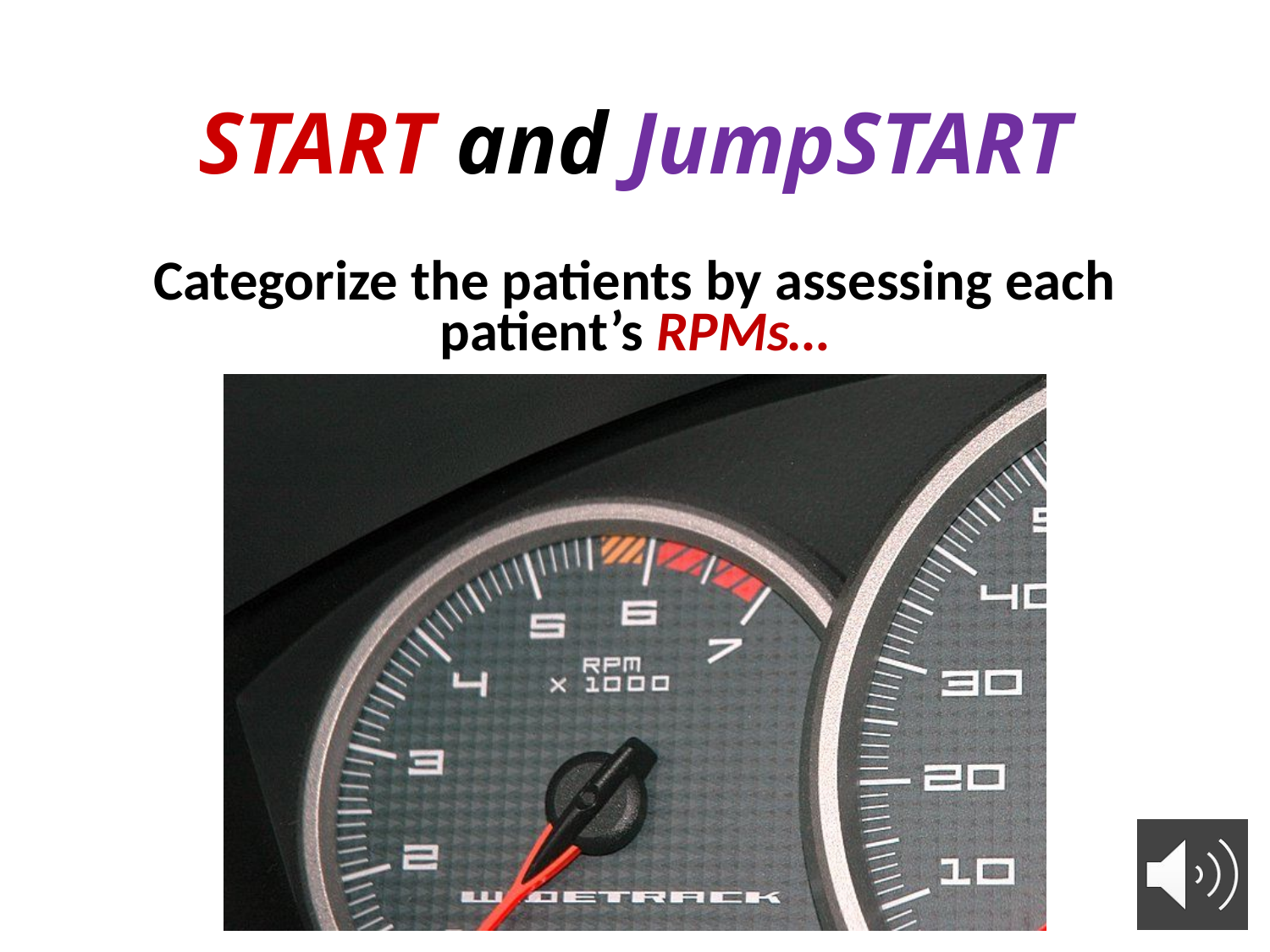

# START and JumpSTART
Categorize the patients by assessing each patient’s RPMs…

## Slide 13
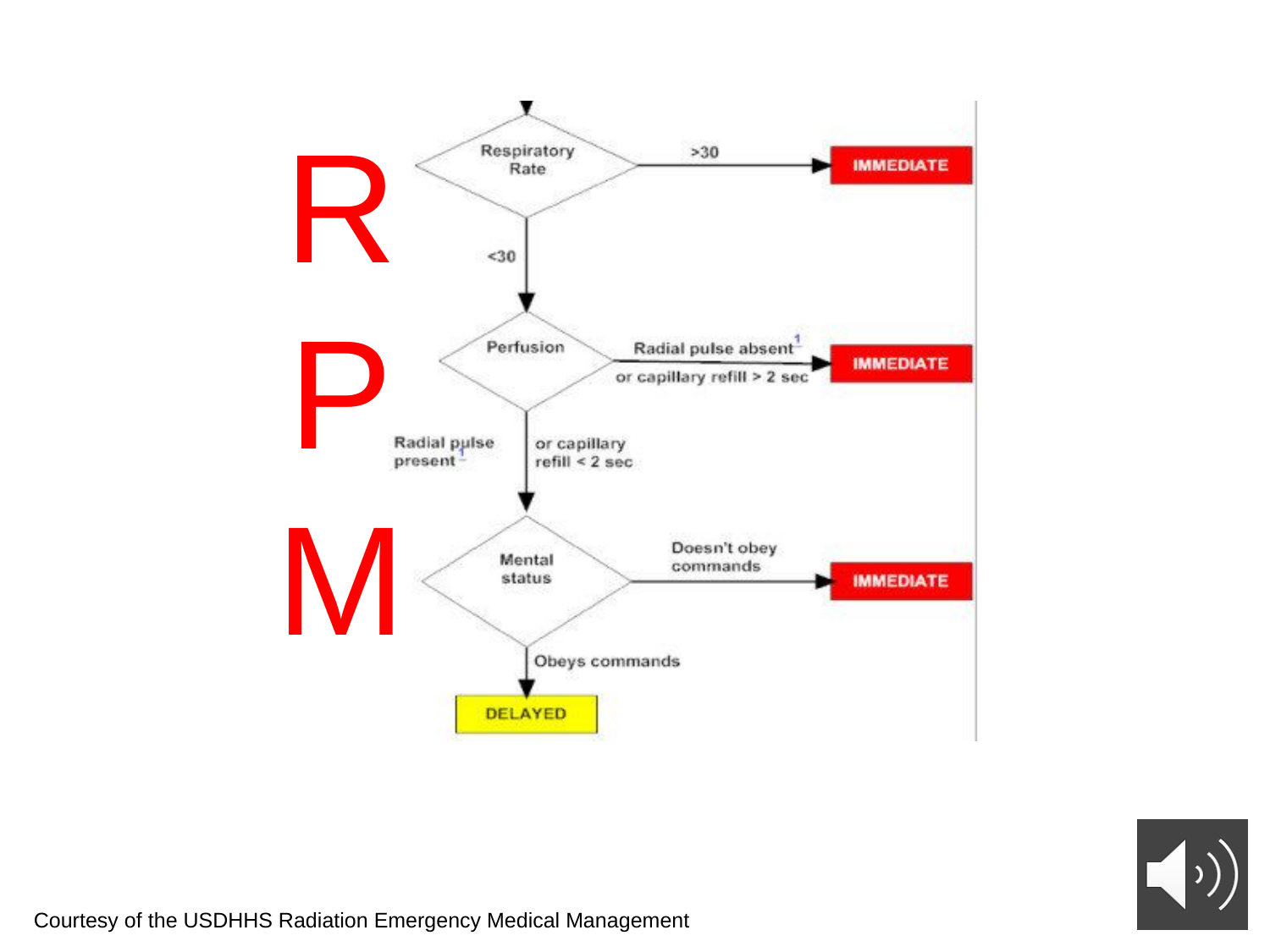

R
P
M
Courtesy of the USDHHS Radiation Emergency Medical Management

## Slide 14
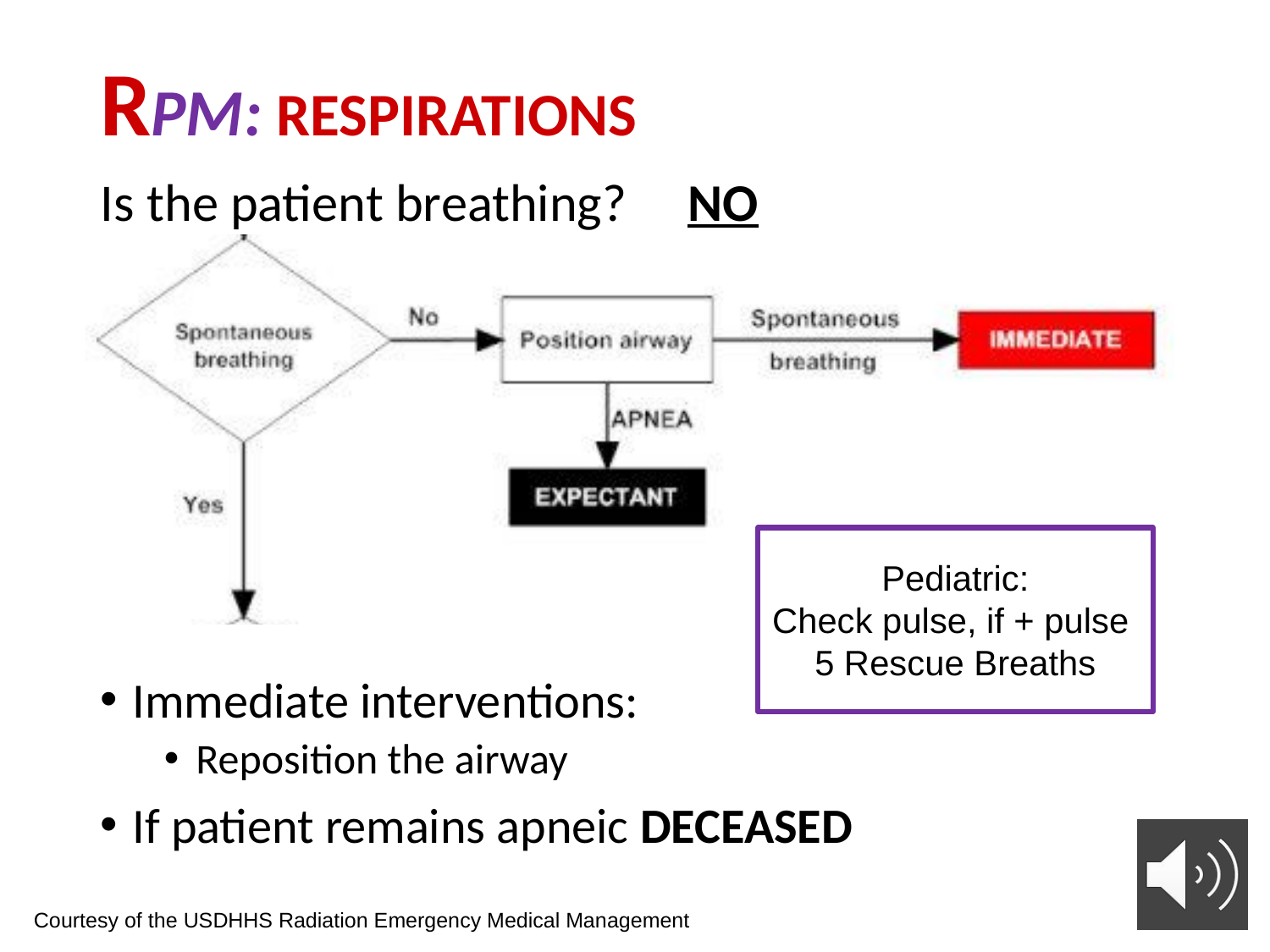

# RPM: RESPIRATIONS
Is the patient breathing? NO
Immediate interventions:
Reposition the airway
If patient remains apneic DECEASED
Pediatric:
Check pulse, if + pulse
5 Rescue Breaths
Courtesy of the USDHHS Radiation Emergency Medical Management

## Slide 15
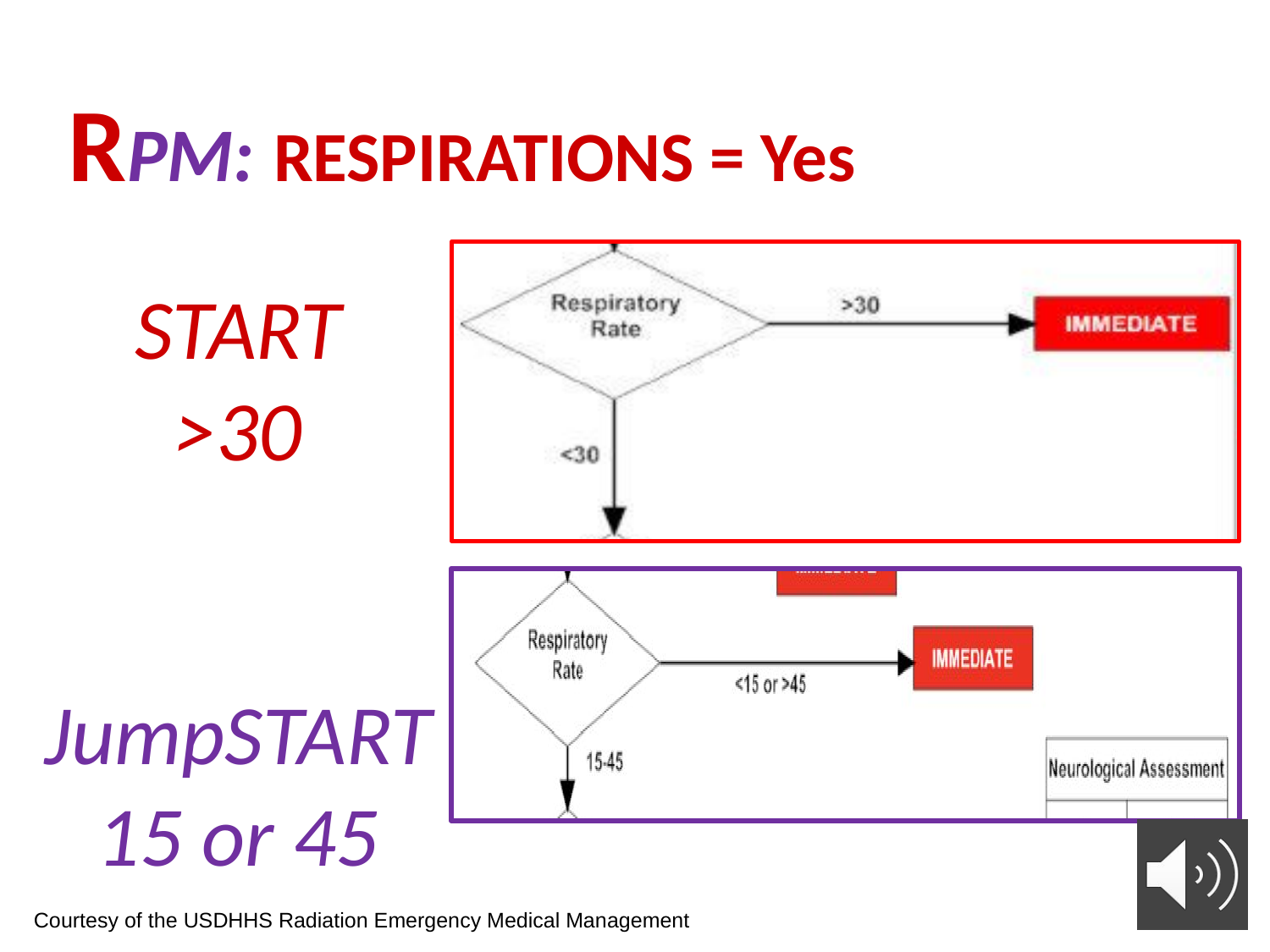

# RPM: RESPIRATIONS = Yes
START
>30
JumpSTART
15 or 45
Courtesy of the USDHHS Radiation Emergency Medical Management

## Slide 16
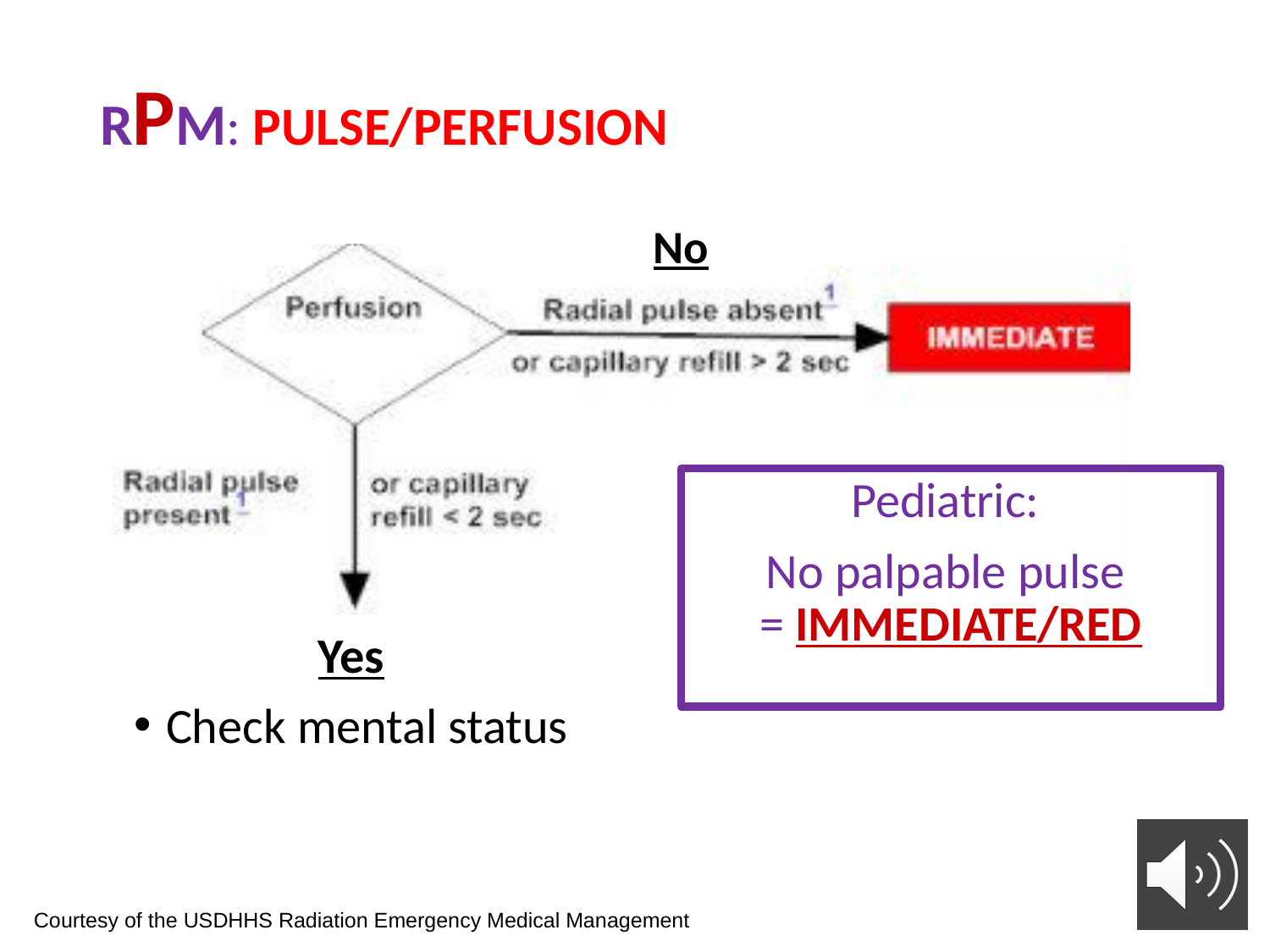

# RPM: PULSE/PERFUSION
No
Pediatric:
No palpable pulse
= IMMEDIATE/RED
Yes
Check mental status
Courtesy of the USDHHS Radiation Emergency Medical Management

## Slide 17
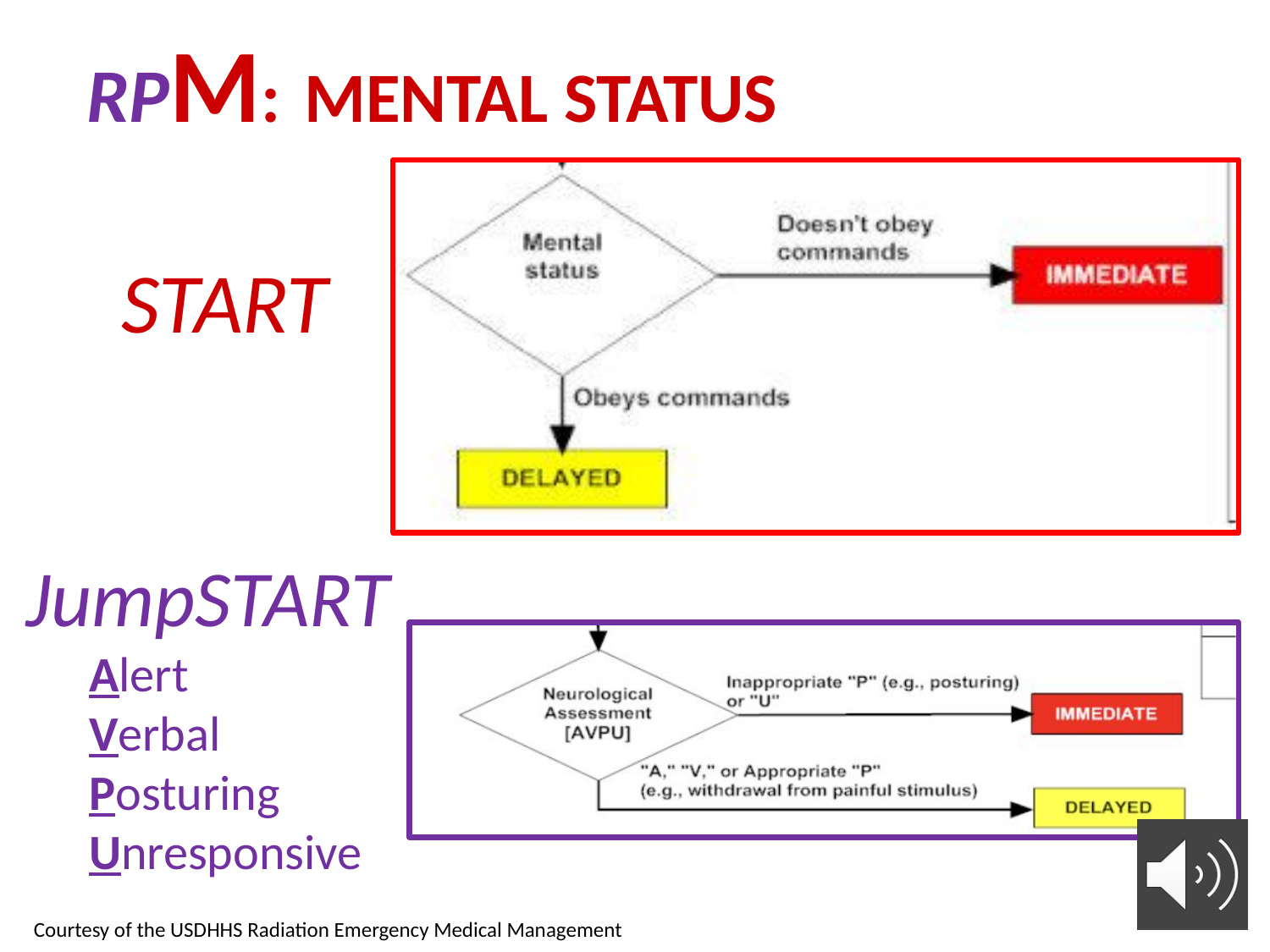

RPM: MENTAL STATUS
START
JumpSTART
Alert
Verbal
Posturing
Unresponsive
Courtesy of the USDHHS Radiation Emergency Medical Management

## Slide 18
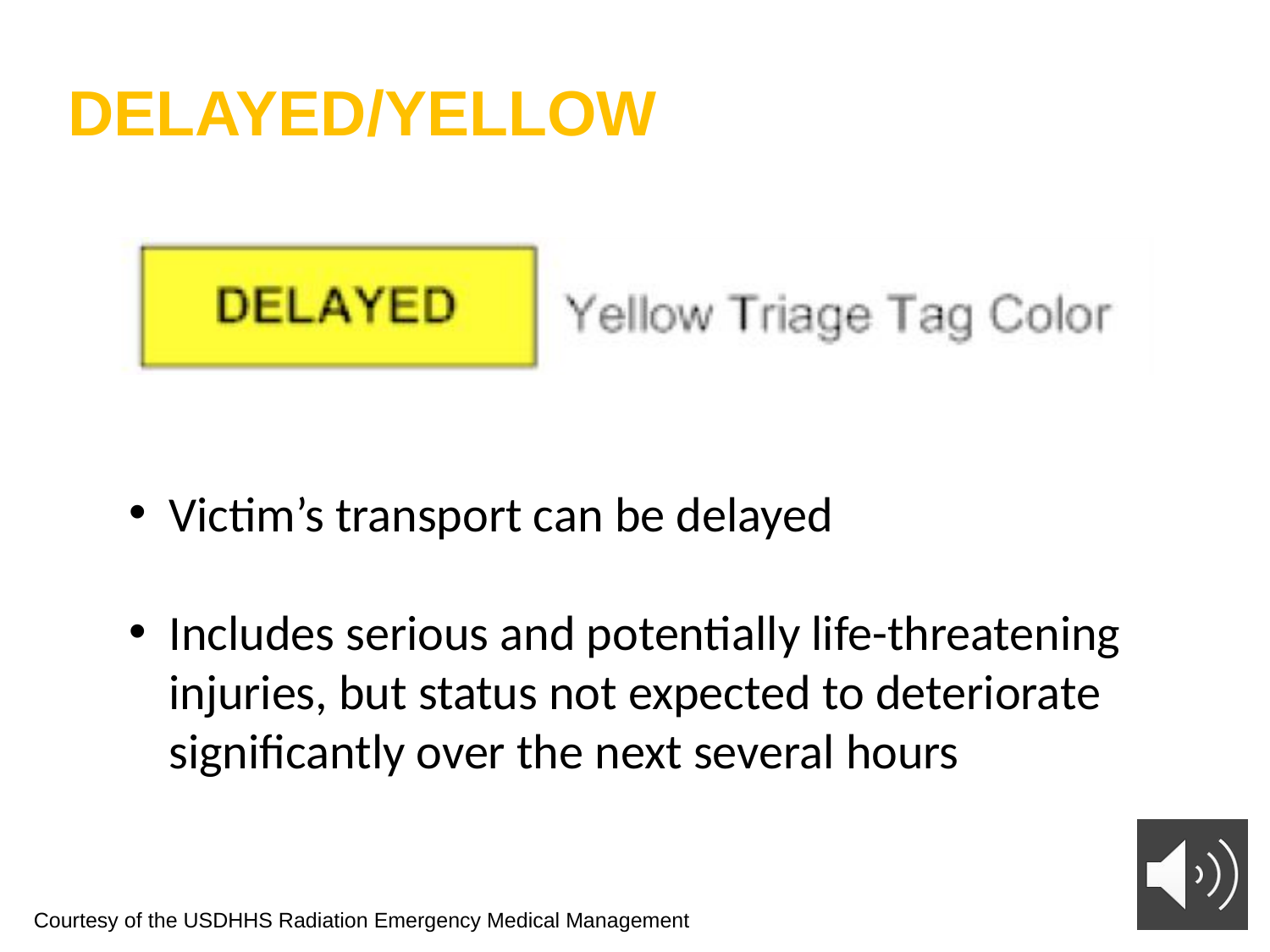

# DELAYED/YELLOW
Victim’s transport can be delayed
Includes serious and potentially life-threatening injuries, but status not expected to deteriorate significantly over the next several hours
Courtesy of the USDHHS Radiation Emergency Medical Management

## Slide 19
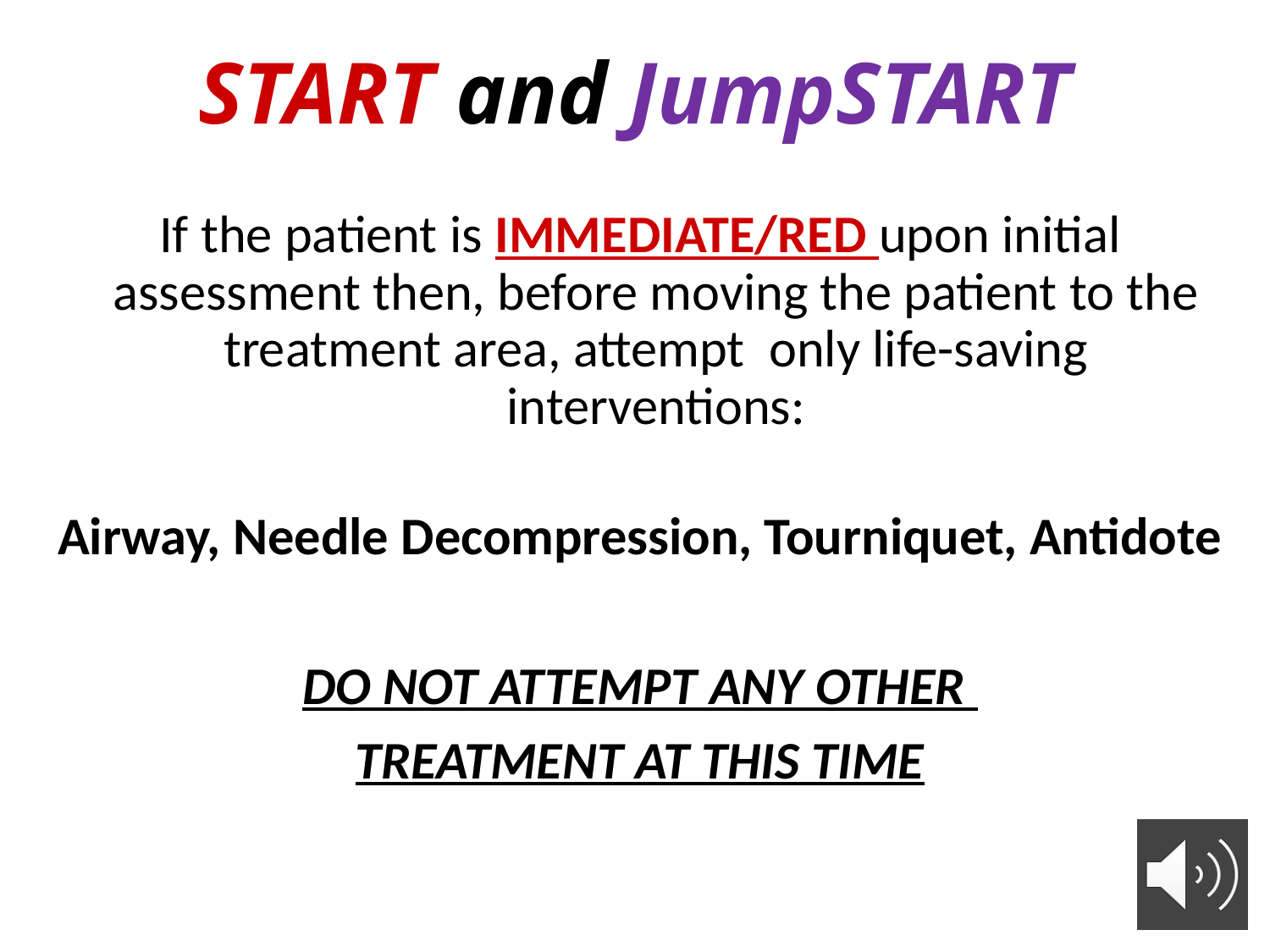

START and JumpSTART
If the patient is IMMEDIATE/RED upon initial assessment then, before moving the patient to the treatment area, attempt only life-saving interventions:
Airway, Needle Decompression, Tourniquet, Antidote
DO NOT ATTEMPT ANY OTHER
TREATMENT AT THIS TIME

## Slide 20
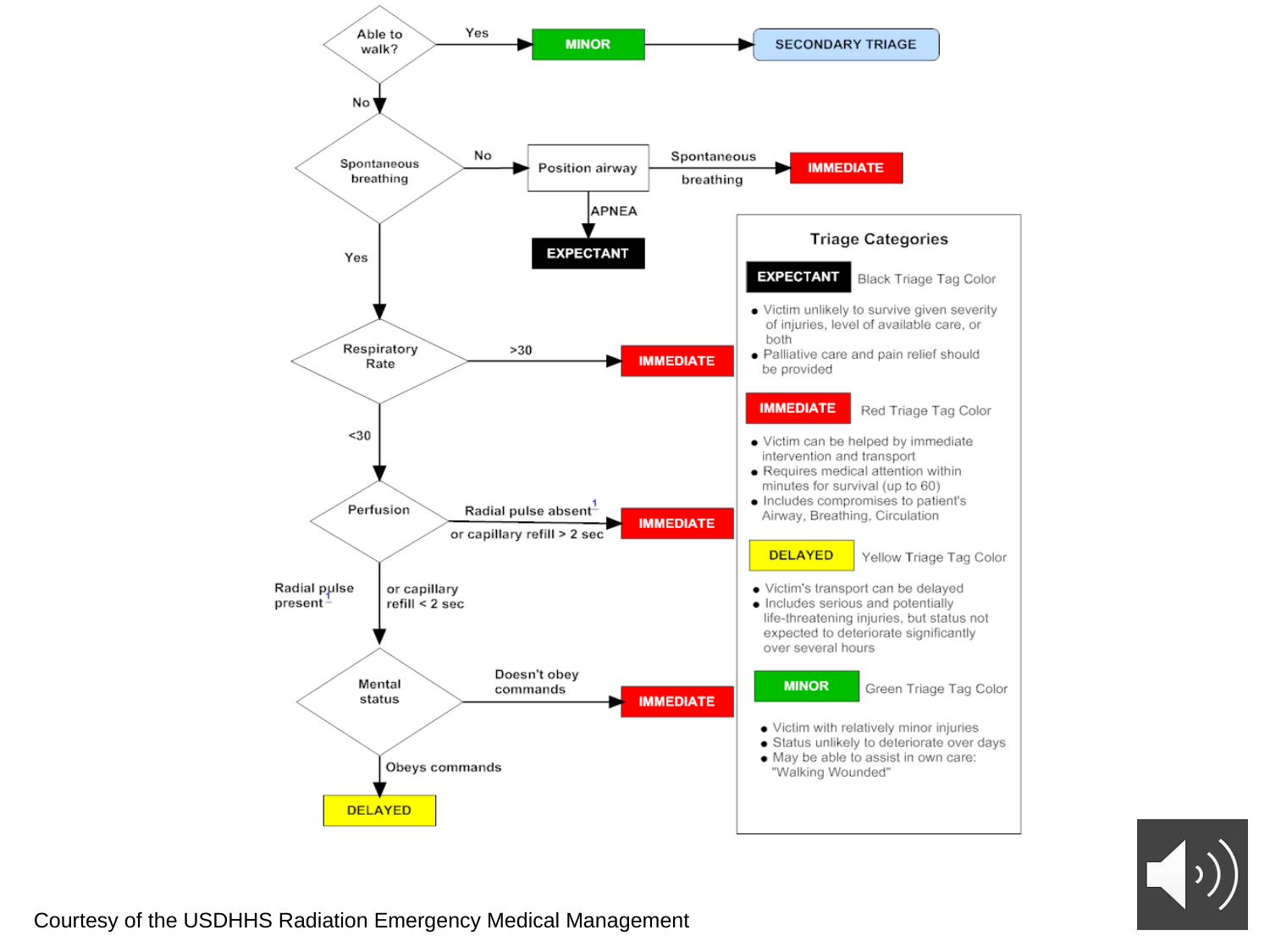

Courtesy of the USDHHS Radiation Emergency Medical Management

## Slide 21
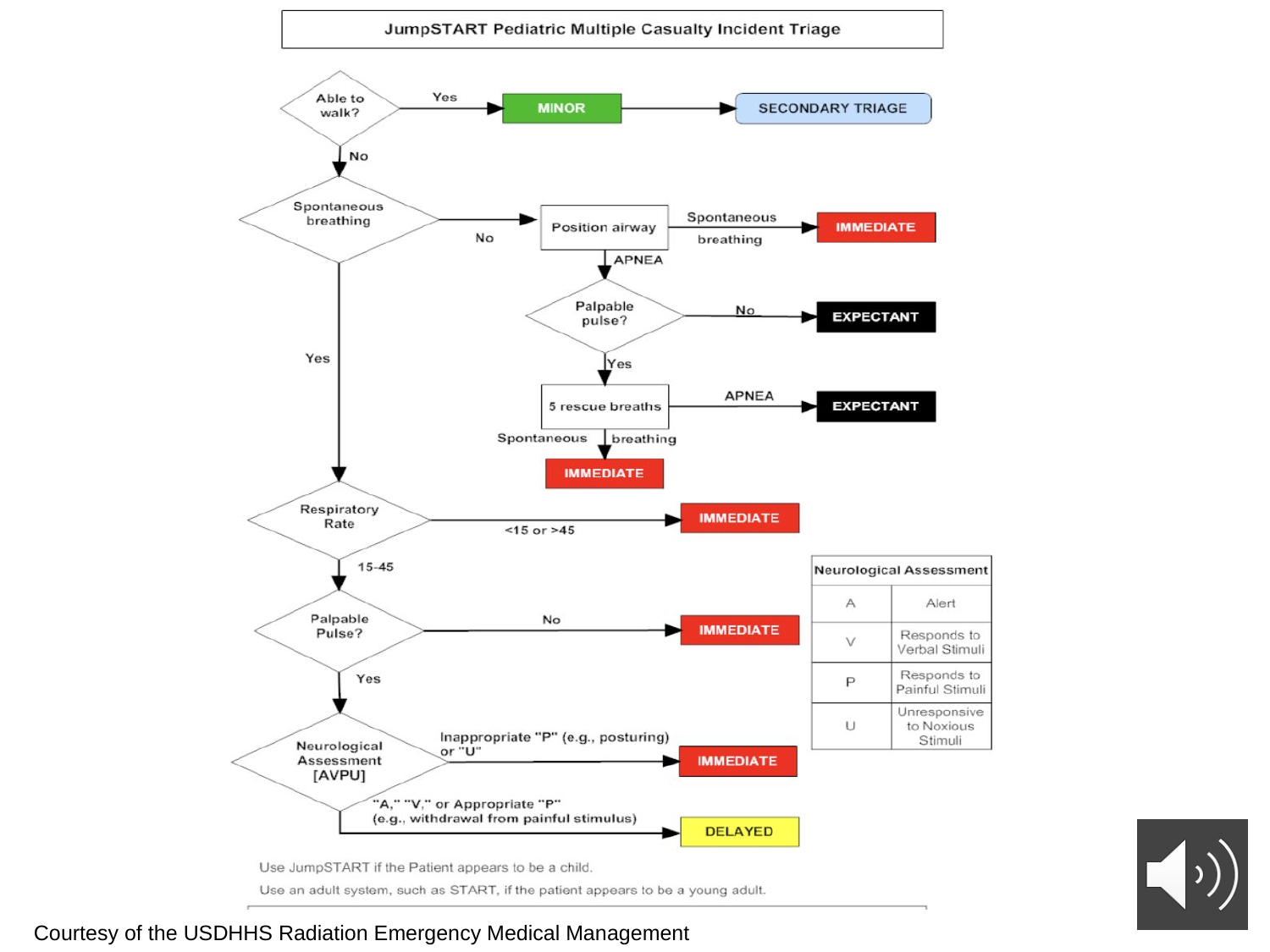

Courtesy of the USDHHS Radiation Emergency Medical Management

## Slide 22
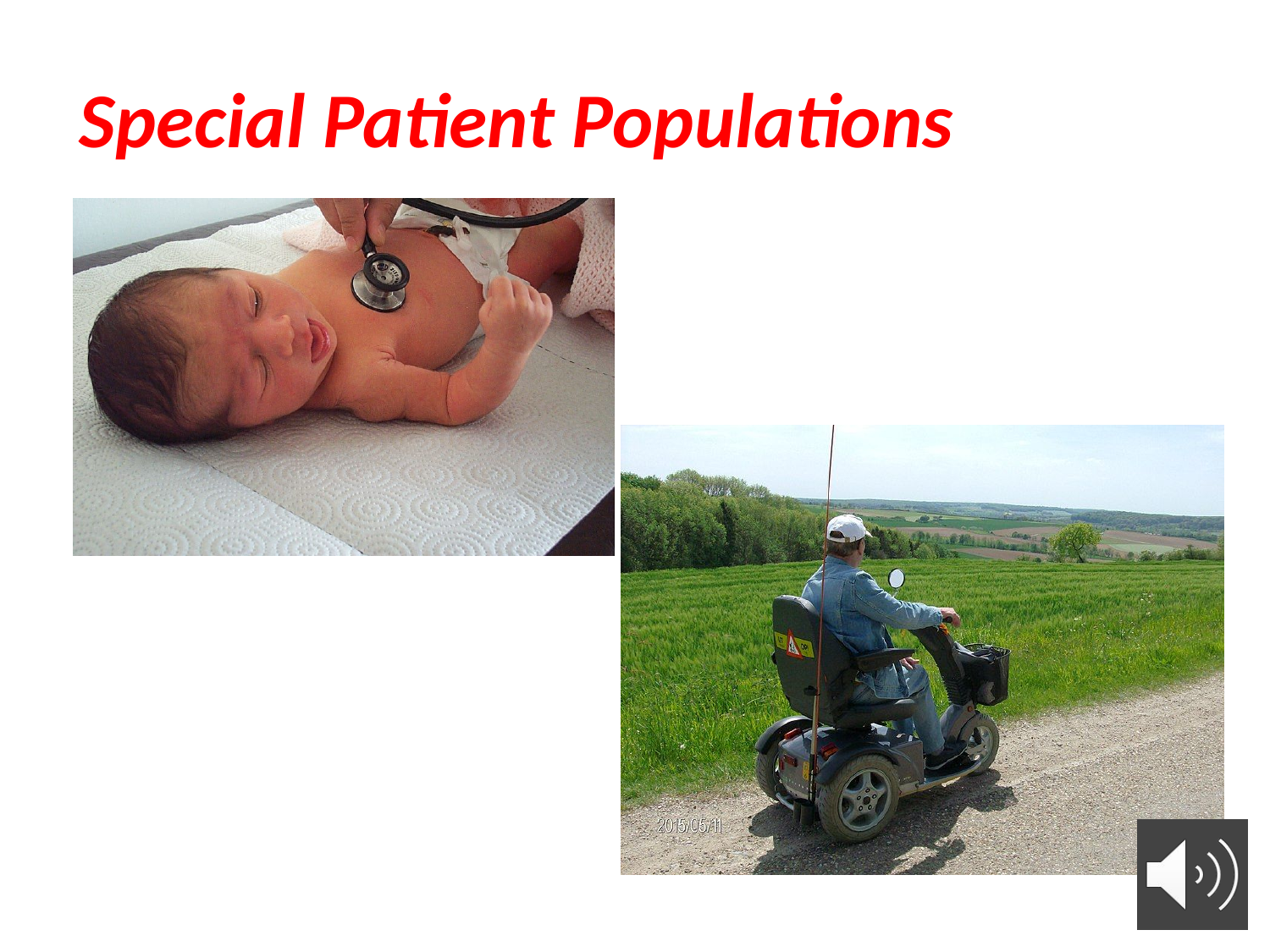

# Special Patient Populations

## Slide 23
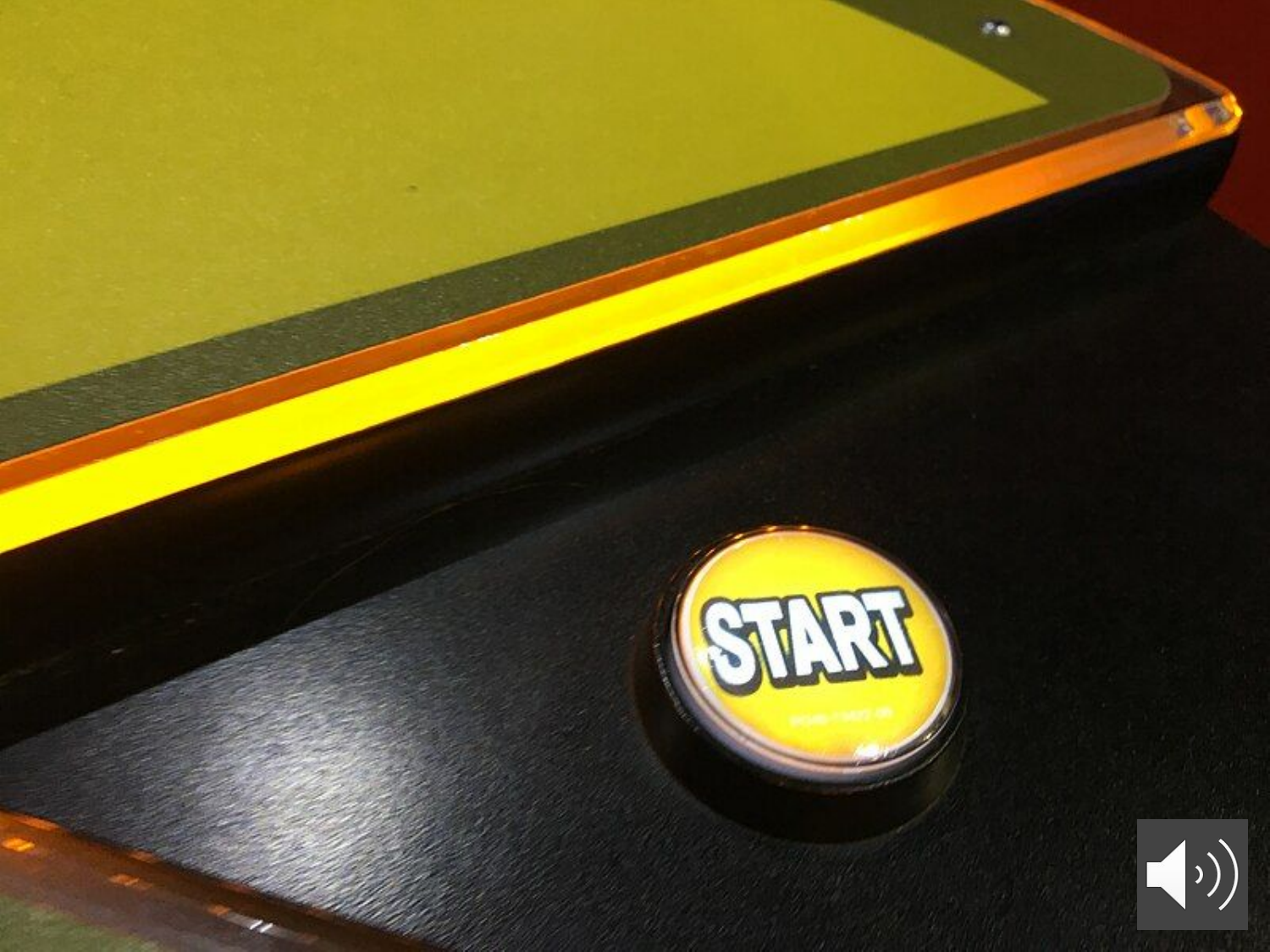

## Slide 24
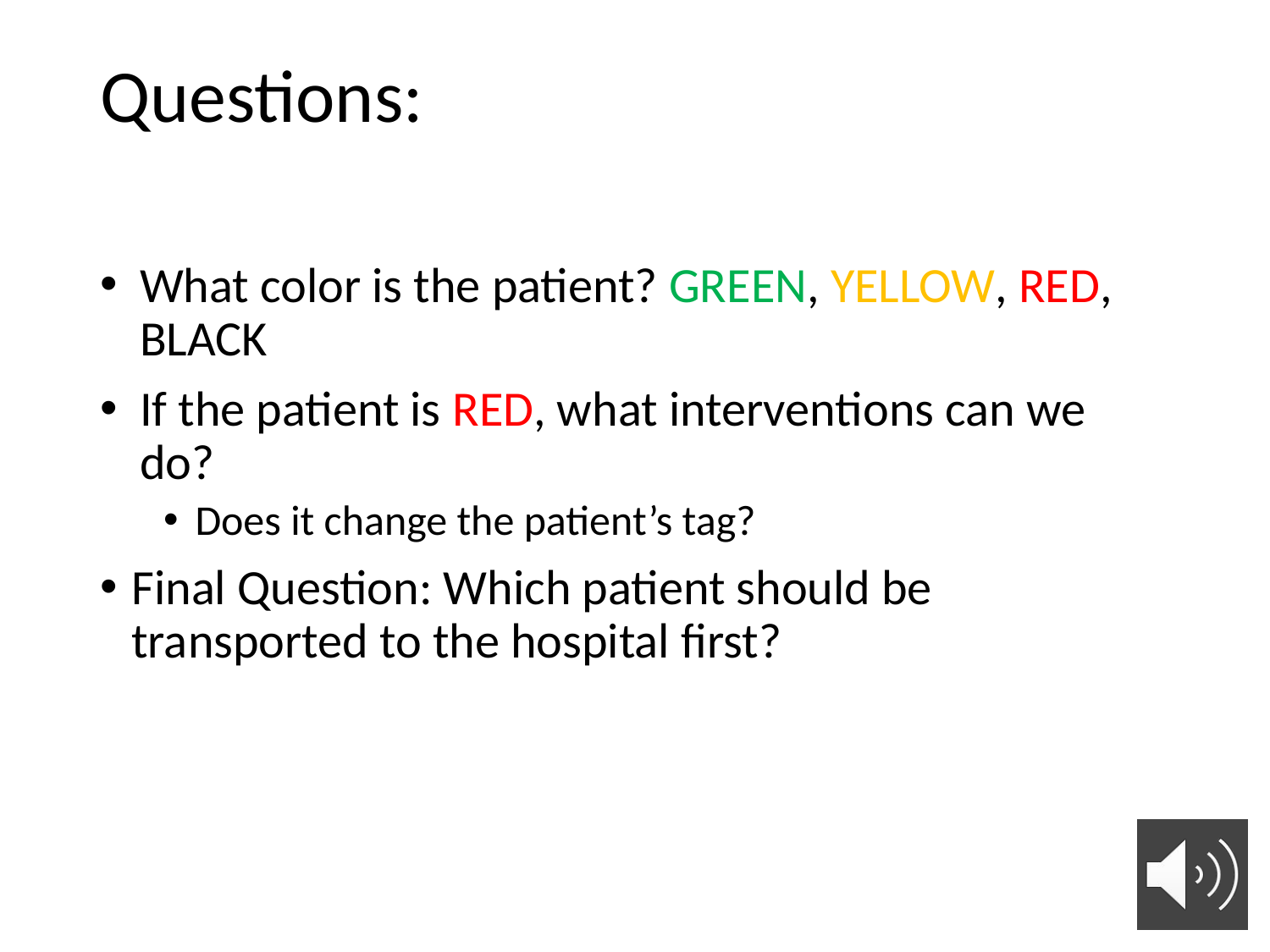

# Questions:
What color is the patient? GREEN, YELLOW, RED, BLACK
If the patient is RED, what interventions can we do?
Does it change the patient’s tag?
Final Question: Which patient should be transported to the hospital first?

## Slide 25
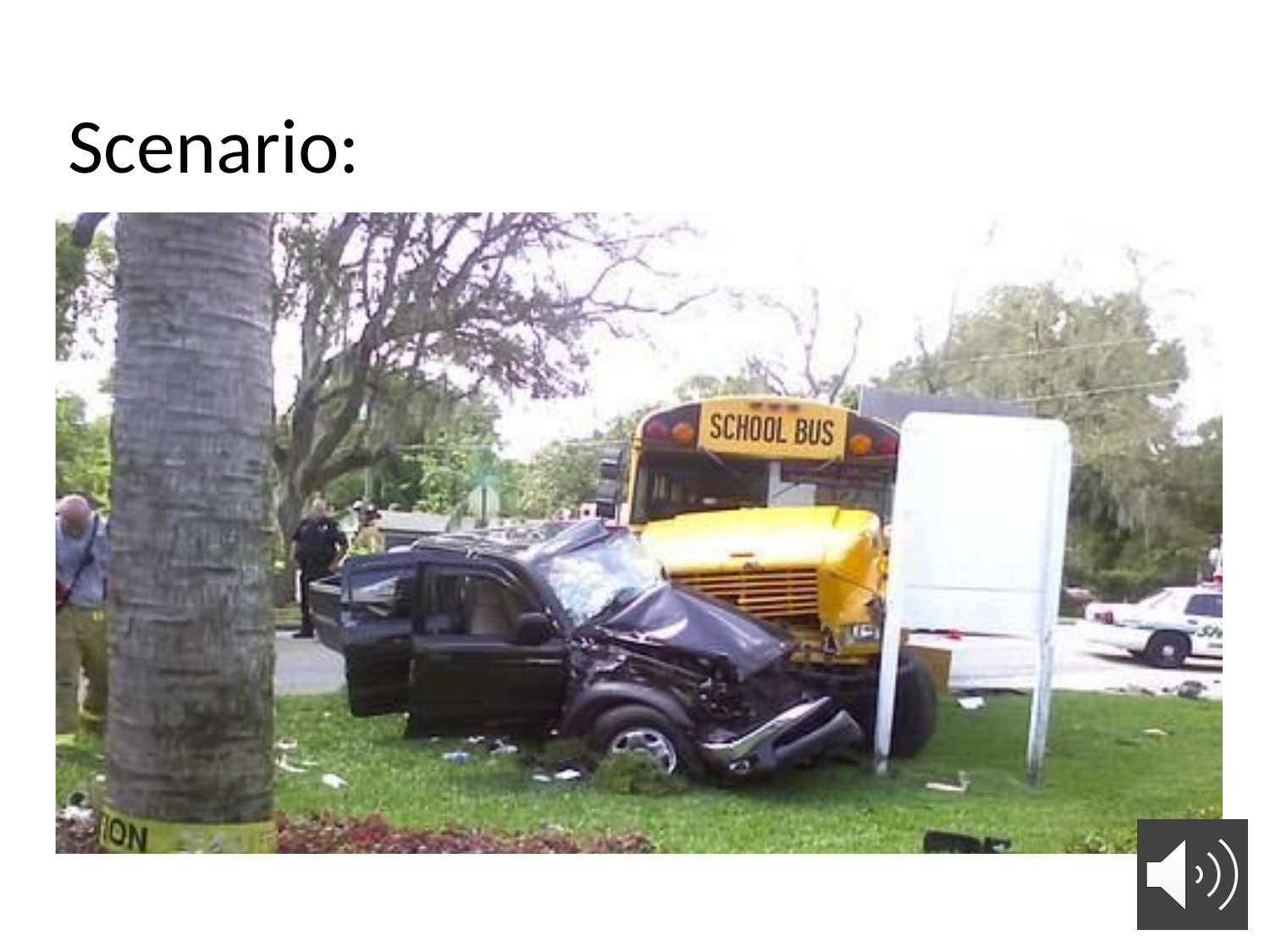

# Scenario:

## Slide 26
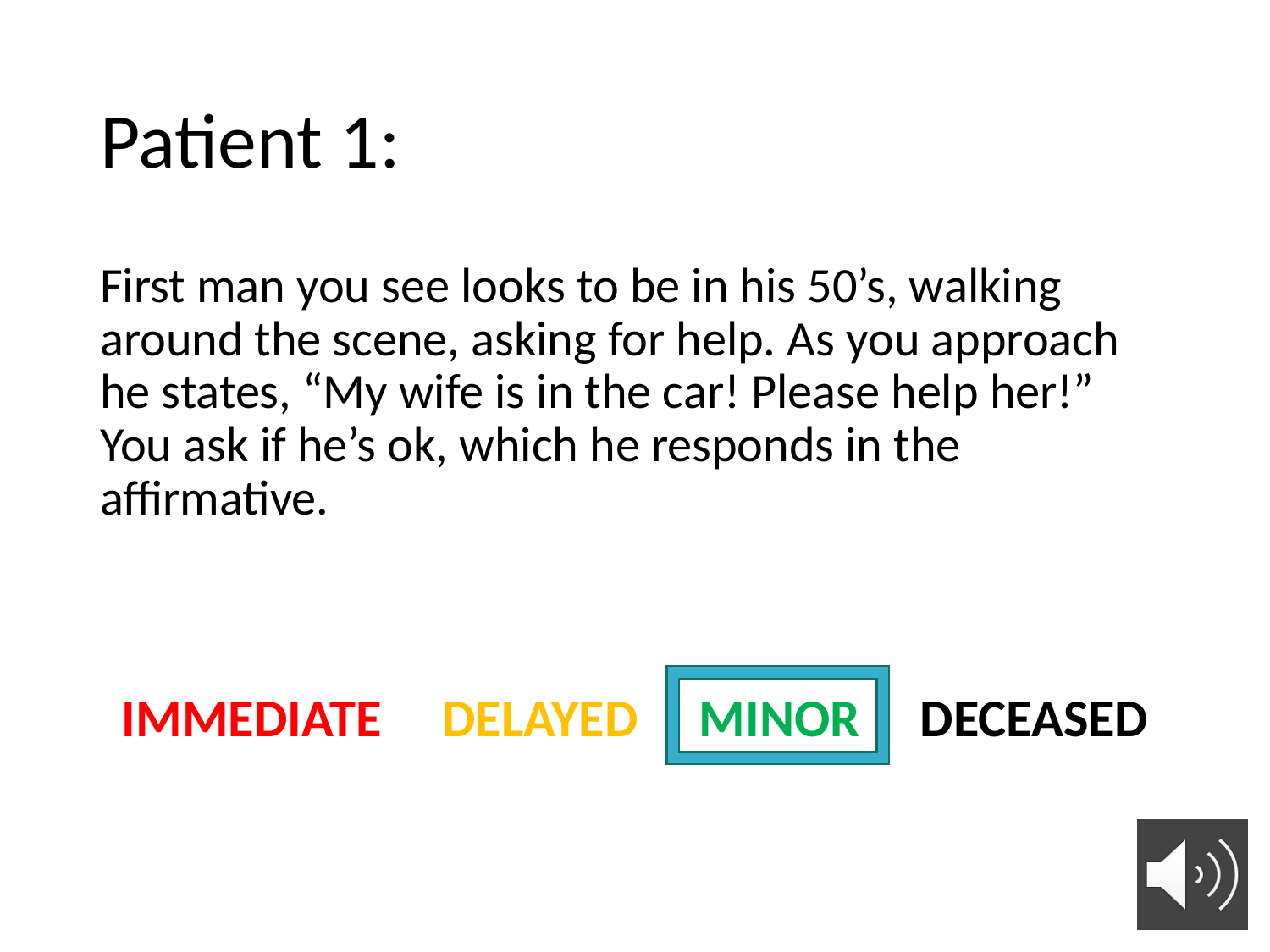

# Patient 1:
First man you see looks to be in his 50’s, walking around the scene, asking for help. As you approach he states, “My wife is in the car! Please help her!” You ask if he’s ok, which he responds in the affirmative.
IMMEDIATE DELAYED MINOR DECEASED

## Slide 27
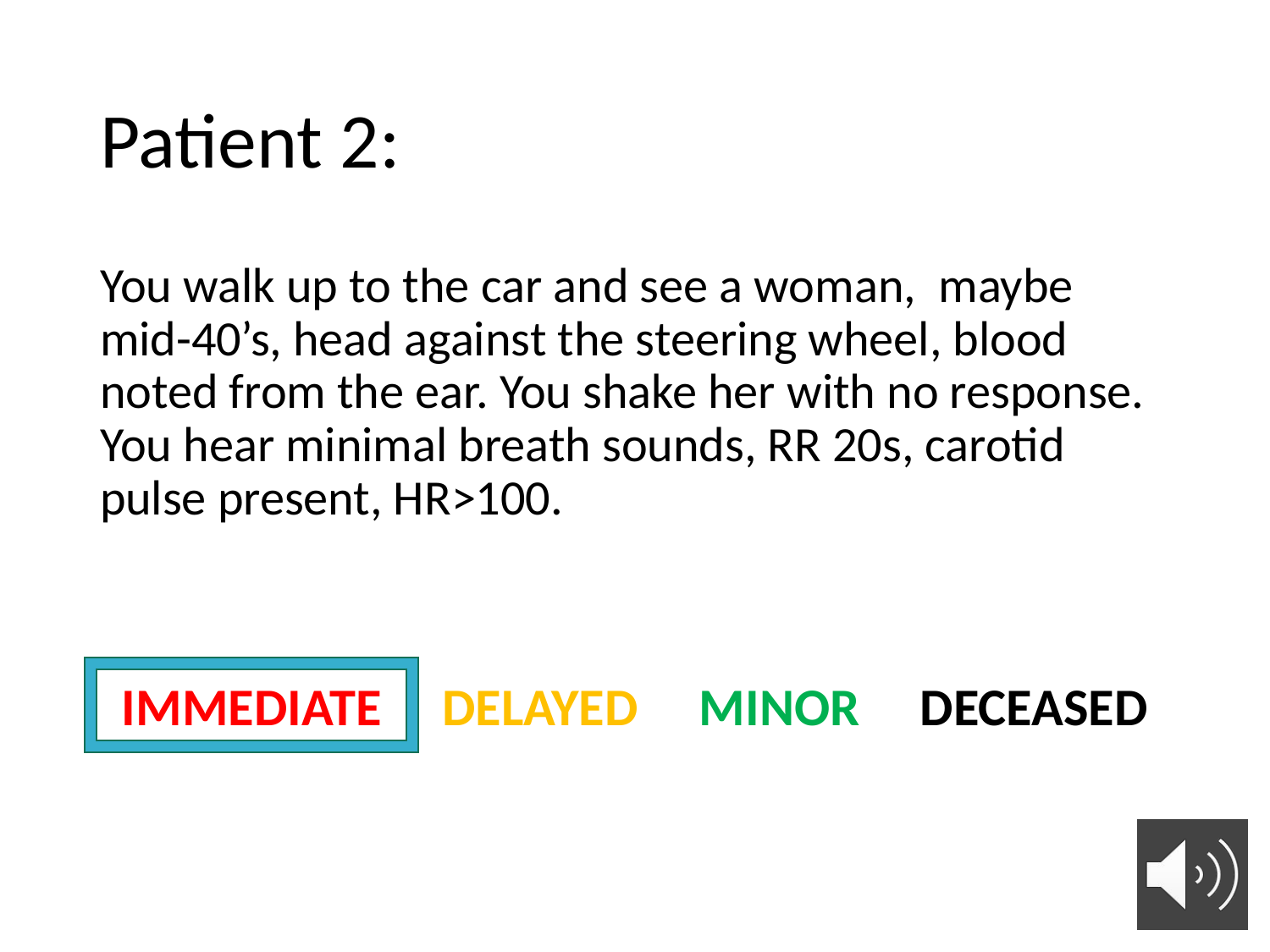

# Patient 2:
You walk up to the car and see a woman, maybe mid-40’s, head against the steering wheel, blood noted from the ear. You shake her with no response. You hear minimal breath sounds, RR 20s, carotid pulse present, HR>100.
IMMEDIATE DELAYED MINOR DECEASED

## Slide 28
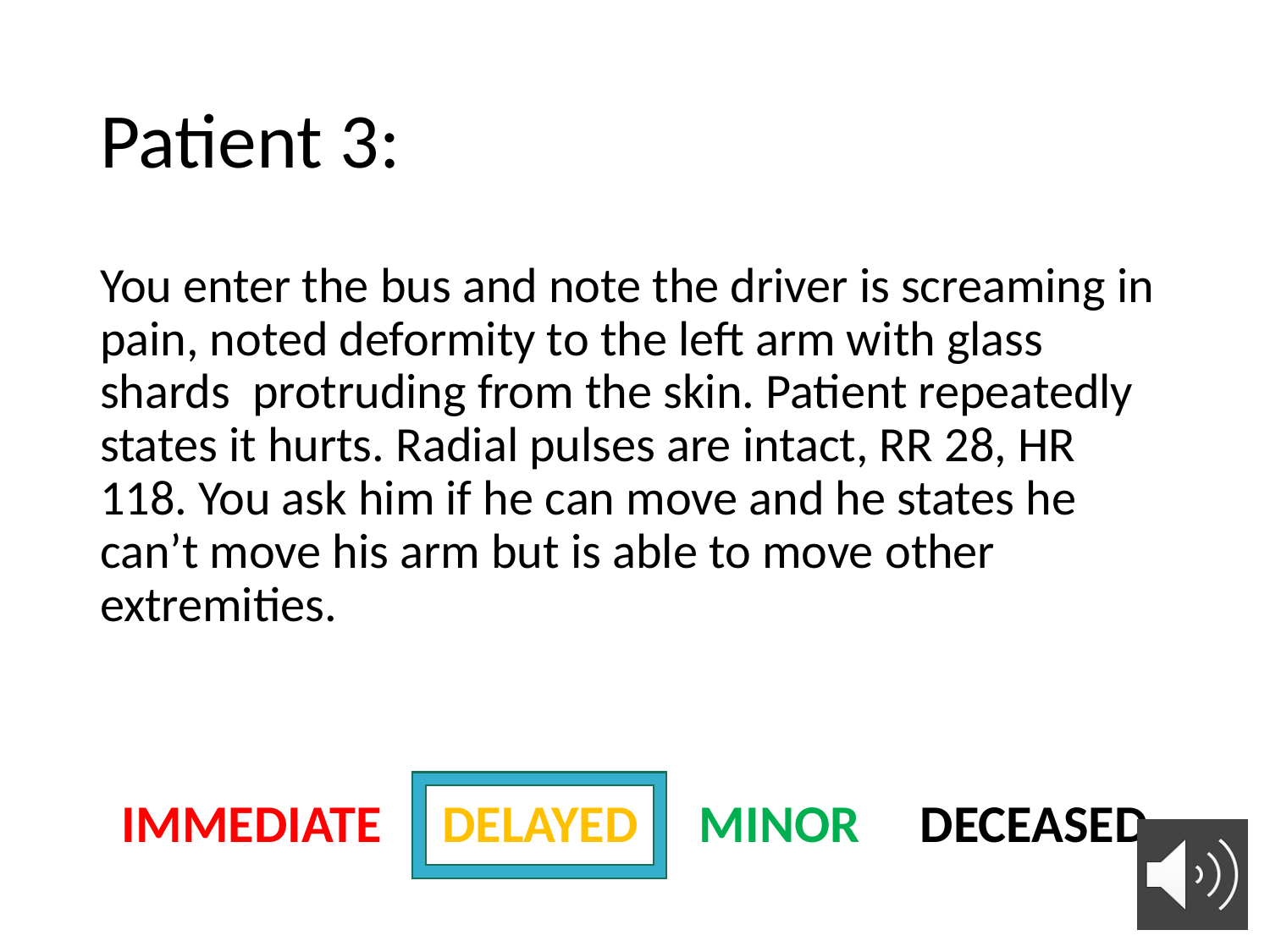

# Patient 3:
You enter the bus and note the driver is screaming in pain, noted deformity to the left arm with glass shards protruding from the skin. Patient repeatedly states it hurts. Radial pulses are intact, RR 28, HR 118. You ask him if he can move and he states he can’t move his arm but is able to move other extremities.
IMMEDIATE DELAYED MINOR DECEASED

## Slide 29
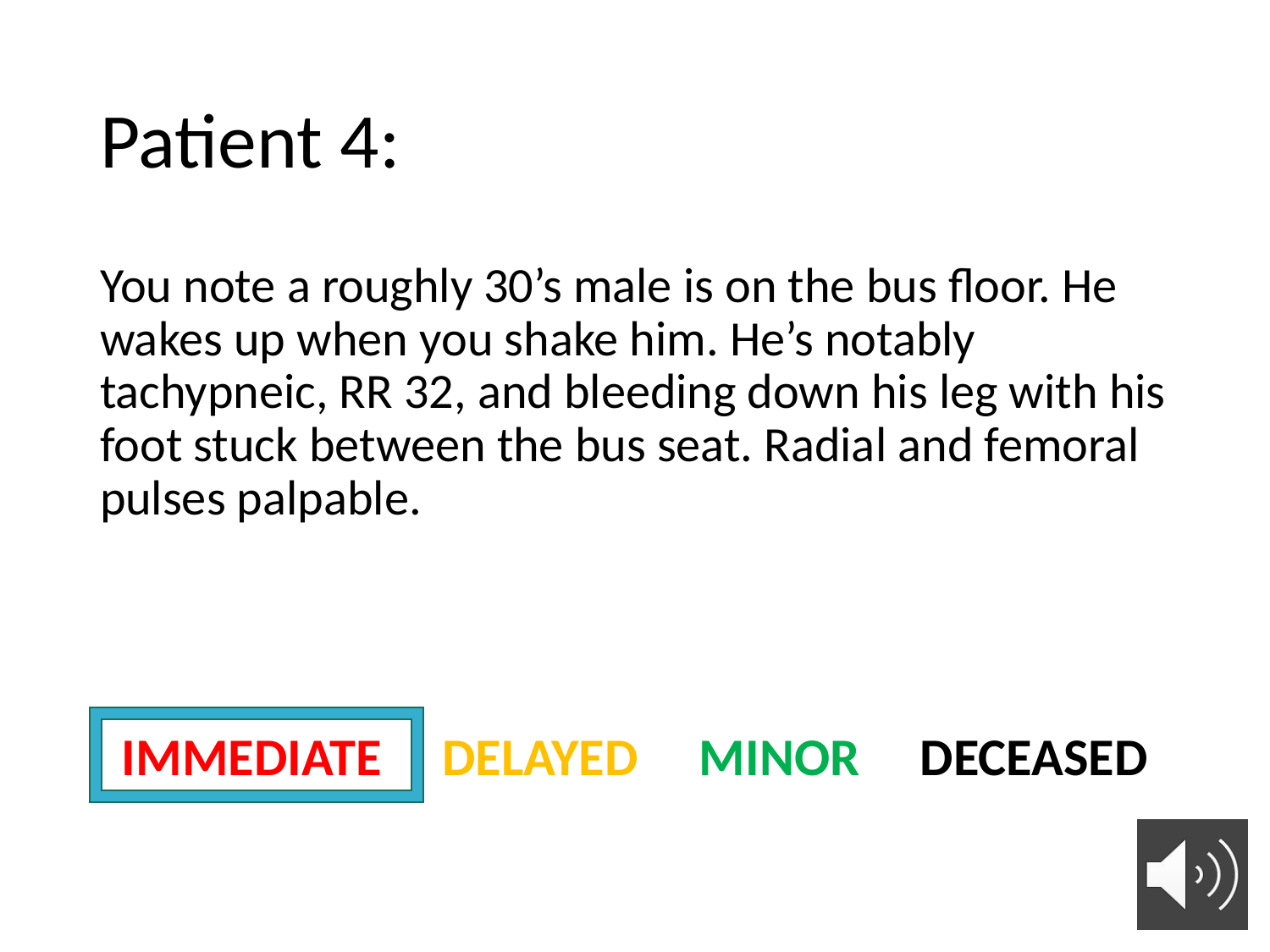

# Patient 4:
You note a roughly 30’s male is on the bus floor. He wakes up when you shake him. He’s notably tachypneic, RR 32, and bleeding down his leg with his foot stuck between the bus seat. Radial and femoral pulses palpable.
IMMEDIATE DELAYED MINOR DECEASED

## Slide 30
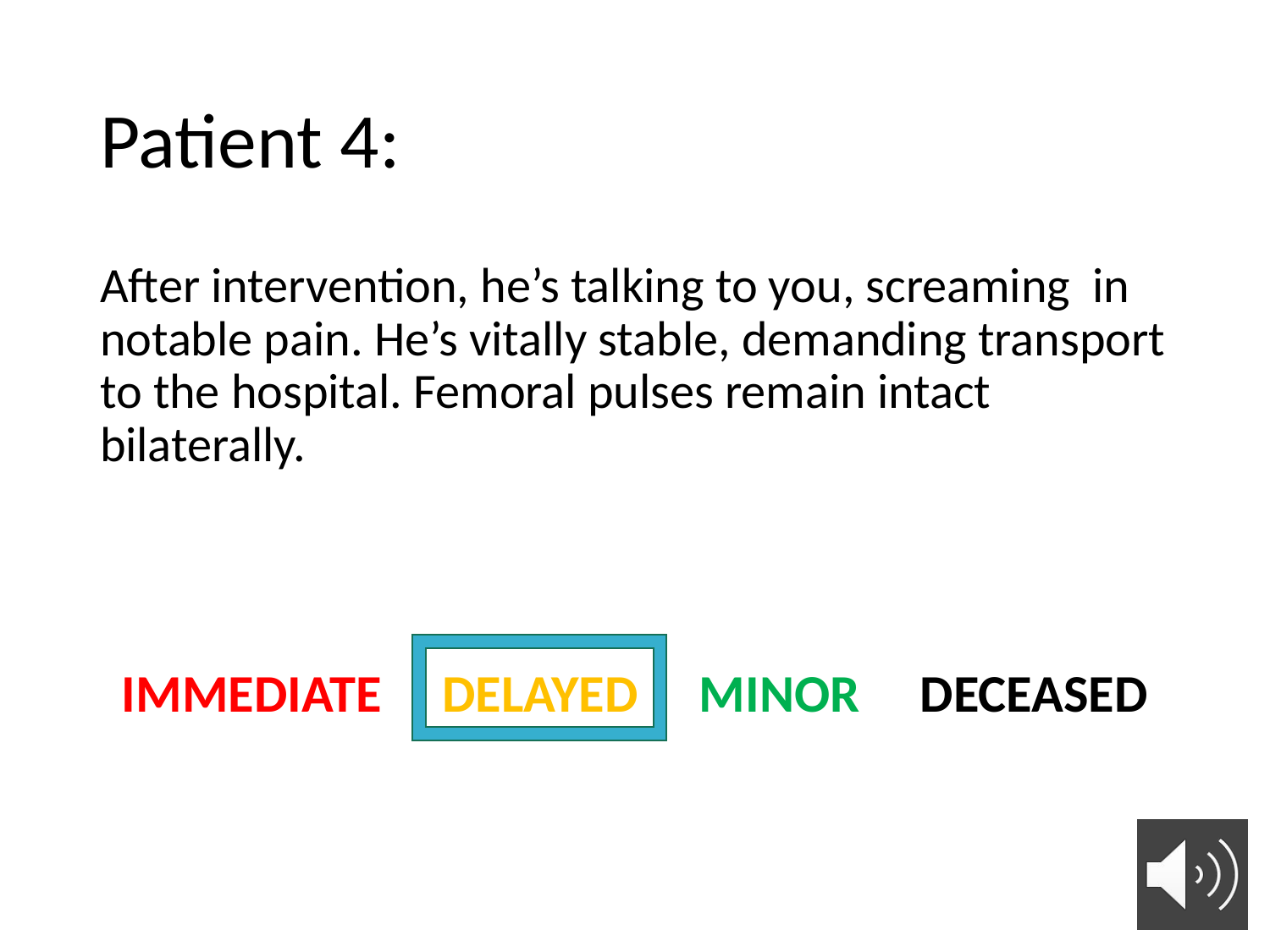

# Patient 4:
After intervention, he’s talking to you, screaming in notable pain. He’s vitally stable, demanding transport to the hospital. Femoral pulses remain intact bilaterally.
IMMEDIATE DELAYED MINOR DECEASED

## Slide 31
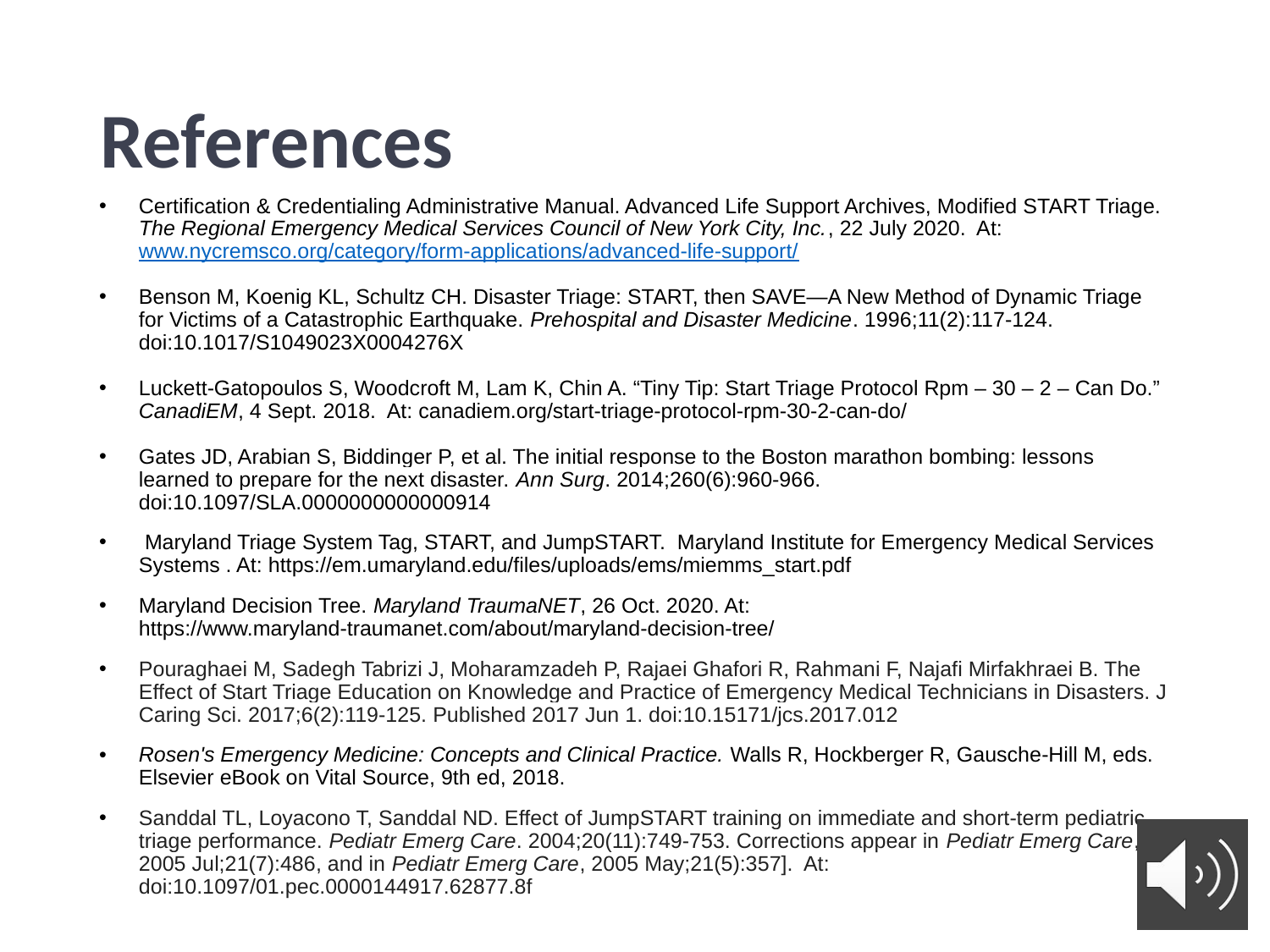

# References
Certification & Credentialing Administrative Manual. Advanced Life Support Archives, Modified START Triage. The Regional Emergency Medical Services Council of New York City, Inc., 22 July 2020. At: www.nycremsco.org/category/form-applications/advanced-life-support/
Benson M, Koenig KL, Schultz CH. Disaster Triage: START, then SAVE—A New Method of Dynamic Triage for Victims of a Catastrophic Earthquake. Prehospital and Disaster Medicine. 1996;11(2):117-124. doi:10.1017/S1049023X0004276X
Luckett-Gatopoulos S, Woodcroft M, Lam K, Chin A. “Tiny Tip: Start Triage Protocol Rpm – 30 – 2 – Can Do.” CanadiEM, 4 Sept. 2018. At: canadiem.org/start-triage-protocol-rpm-30-2-can-do/
Gates JD, Arabian S, Biddinger P, et al. The initial response to the Boston marathon bombing: lessons learned to prepare for the next disaster. Ann Surg. 2014;260(6):960-966. doi:10.1097/SLA.0000000000000914
 Maryland Triage System Tag, START, and JumpSTART. Maryland Institute for Emergency Medical Services Systems . At: https://em.umaryland.edu/files/uploads/ems/miemms_start.pdf
Maryland Decision Tree. Maryland TraumaNET, 26 Oct. 2020. At: https://www.maryland-traumanet.com/about/maryland-decision-tree/
Pouraghaei M, Sadegh Tabrizi J, Moharamzadeh P, Rajaei Ghafori R, Rahmani F, Najafi Mirfakhraei B. The Effect of Start Triage Education on Knowledge and Practice of Emergency Medical Technicians in Disasters. J Caring Sci. 2017;6(2):119-125. Published 2017 Jun 1. doi:10.15171/jcs.2017.012
Rosen's Emergency Medicine: Concepts and Clinical Practice. Walls R, Hockberger R, Gausche-Hill M, eds. Elsevier eBook on Vital Source, 9th ed, 2018.
Sanddal TL, Loyacono T, Sanddal ND. Effect of JumpSTART training on immediate and short-term pediatric triage performance. Pediatr Emerg Care. 2004;20(11):749-753. Corrections appear in Pediatr Emerg Care, 2005 Jul;21(7):486, and in Pediatr Emerg Care, 2005 May;21(5):357].  At: doi:10.1097/01.pec.0000144917.62877.8f
